# Molecular mechanisms, targets and clinical potential of berberine in regulating metabolism: a review focussing on databases and molecular docking studies

**DOI:** 10.3389/fphar.2024.1368950

**Published:** 2024-06-18

**Authors:** Aru Sun, Haoyu Yang, Tao Li, Jinli Luo, Ling Zhou, Rui Chen, Lin Han, Yiqun Lin

**Affiliations:** ^1^ Institute of Metabolic Diseases, Guang’anmen Hospital, China Academy of Chinese Medical Sciences, Beijing, China; ^2^ College of Traditional Chinese Medicine, Changchun University of Chinese Medicine, Changchun, China; ^3^ China Traditional Chinese Medicine Holdings Co. Limited, Guangdong e-fong Pharmaceutical Co., Ltd., Foshan, China; ^4^ Graduate College, Beijing University of Chinese Medicine, Beijing, China; ^5^ College of Basic Medical Sciences, Changchun University of Chinese Medicine, Changchun, China; ^6^ Department of Endocrinology, Guang’anmen Hospital South Campus, China Academy of Chinese Medical Sciences, Beijing, China

**Keywords:** berberine, metabolism, potential targets, signaling pathway, molecular docking

## Abstract

**Background:** Metabolic imbalance is the common basis of many diseases. As natural isoquinoline alkaloid, berberine (BBR) has shown great promise in regulating glucose and lipids metabolism and treating metabolic disorders. However, the related mechanism still lacks systematic research.

**Aim:** To discuss the role of BBR in the whole body’s systemic metabolic regulation and further explore its therapeutic potential and targets.

**Method:** Based on animal and cell experiments, the mechanism of BBR regulating systemic metabolic processes is reviewed. Potential metabolism-related targets were summarized using Therapeutic Target Database (TTD), DrugBank, GeneCards, and cutting-edge literature. Molecular modeling was applied to explore BBR binding to the potential targets.

**Results:** BBR regulates the whole-body metabolic response including digestive, circulatory, immune, endocrine, and motor systems through adenosine 5’-monophosphate (AMP)-activated protein kinase (AMPK)/mammalian target of rapamycin (mTOR), sirtuin (SIRT)1/forkhead box O (FOXO)1/sterol regulatory element-binding protein (SREBP)2, nuclear factor erythroid 2-related factor (Nrf) 2/heme oxygenase (HO)-1, and other signaling pathways. Through these reactions, BBR exerts hypoglycemic, lipid-regulating, anti-inflammatory, anti-oxidation, and immune regulation. Molecular docking results showed that BBR could regulate metabolism targeting FOXO3, Nrf2, NAD(P)H quinone oxidoreductase 1 (NQO1), glutathione peroxidase (Gpx) 4 and phosphatidylinositol-4,5-bisphosphate 3-kinase catalytic subunit alpha (PIK3CA). Evaluating the target clinical effects, we found that BBR has the therapeutic potential of anti-aging, anti-cancer, relieving kidney disease, regulating the nervous system, and alleviating other chronic diseases.

**Conclusion:** This review elucidates the interaction between potential targets and small molecular metabolites by exploring the mechanism of BBR regulating metabolism. That will help pharmacologists to identify new promising metabolites interacting with these targets.

## 1 Introduction

The life of the human body depends on the normal operation of metabolism. If metabolism cannot function normally, it can lead to different degrees of physiological abnormalities and to diseases. Metabolic diseases, such as type 2 diabetes, obesity, and non-alcoholic fatty liver disease, have become increasingly serious global health challenges due to the sharp increase in their incidence (Ling et al., 2023). As of 2021, the estimated prevalence of diabetes among adults aged 20–79 worldwide is 536.6 million, of which 90% are type 2 diabetes. Long-term hyperglycemia can lead to multiple complications, including vision loss, kidney disease and neuropathy, affecting patients’ quality of life (Sun et al., 2022). About one-third of the world’s population suffers from non-alcoholic fatty liver disease, and some non-alcoholic fatty liver disease patients will further develop liver cirrhosis or hepatocellular carcinoma, threatening their lives (Wong et al., 2023). Obesity increases the risk of patients suffering from heart disease, diabetes, sleep apnea, joint disease and certain types of cancer. In addition, obesity can also have an impact on mental health, leading to depression and low self-esteem (Jin et al., 2023). The occurrence of these diseases is related to various factors, including genetics, environment, and lifestyle. However, effective treatment methods are still limited, especially for improving the fundamental causes of metabolic diseases. How to restore the relative balance of metabolism in the body, explore metabolic regulation strategies, and analyze the information exchange mechanisms in these regulatory processes are urgent problems to be solved.

More and more researchers are attempting to find effective drugs for metabolic diseases from natural products. Berberine (BBR) is an isoquinoline alkaloid found in the plant families Ranunculaceae, Berberidaceae, and Papaveraceae, particularly in the Coptis genus (Wong et al., 2020). Medicinal plants containing BBR have been used for more than 3,000 years. In clinical practice, BBR was originally used primarily for the treatment of digestive tract diseases, owing to its antibacterial and anti-inflammatory properties (Song et al., 2020). In recent years, BBR has attracted great interest of researchers due to its extensive pharmacological activities, especially its potential to regulate metabolism. More and more studies have shown that BBR plays a role in body metabolism, by regulating lipid and glucose metabolism, inhibiting liver fat accumulation, and alleviating vascular endothelial injury (Xu et al., 2021). In addition, BBR can also regulate intestinal microbiota, further affecting host metabolism. Therefore, BBR is considered a promising agent for the treatment of metabolism-related diseases. The therapeutic effect of BBR is based on the regulation of various metabolic aspects and pathophysiological processes, involving multiple cellular signaling pathways and action targets. For example, BBR enhances the cell’s utilization of glucose and fat by activating AMP-activated protein kinase (AMPK), thus improving insulin sensitivity and lipid metabolism (Chi et al., 2014). BBR can upregulate the expression of low-density lipoprotein receptors on the surface of liver cells, thereby increasing the clearance of the low-density lipoprotein (LDL) receptor (LDL-R) and reducing the level of LDL cholesterol (LDL-C) in the blood (Kong et al., 2004). However, metabolic diseases can involve multiple systems and organs, including the endocrine system, digestive system, and circulatory system. Although the metabolic regulatory function of BBR has been supported by many studies, further studies are still needed to understand the specific mechanism of how BBR regulates body metabolism and its potential clinical application.

This paper systematically reviews the recent research progress on the regulation of body’s metabolism by BBR, summarizing its mechanism and key targets in the system. It has been found that BBR has definite and extensive effects in regulating blood glucose, blood lipids, insulin resistance, and anti-inflammation. The targets involved in these effects mainly include glucagon-like peptide-1 (GLP1), glucose transporter (GLUT) 2 and mitochondrial pyruvate carrier (MPC) 1. In addition, there are far more pathways and targets for BBR to regulate metabolism. We summarize potential metabolism-related targets using TTD, DrugBank, GeneCards, and cutting-edge literature. Through the molecular docking of BBR and disease targets, more potential metabolic targets have been identified and may play a role in therapeutic effects of BBR, such as forkhead box O (FOXO) 3, nuclear factor erythroid 2-related factor (Nrf) 2, NAD (P) H quinone oxidoreductase 1 (NQO1), glutathione peroxidase (GPx) 4 and phosphatidylinositol-4,5-bisphosphate 3-kinase catalytic subunit alpha (PIK3CA). We thus explored the therapeutic potential of BBR in diseases and found that it has therapeutic effects on aging, cancer and hyperuricemia in addition to metabolism-related diseases.

## 2 BBR achieves metabolic regulation through important target cells in the body system

### 2.1 Digestive system

As an important system for digestion and absorption of nutrients, the digestive system can be regarded as the “big chemical factory” of body’s metabolism, which maintains the source of energy for life activities. In particular, the liver has a strong metabolic capacity, and hepatocytes participate in various metabolic processes such as glucose metabolism, fat metabolism, and protein metabolism. Besides the function of digestion and absorption, the differentiated intestinal cells also play a balanced metabolic role through endocrine regulation and barrier protection.

#### 2.1.1 BBR regulates intestinal cell metabolism

The intestine is an important site for nutrient absorption, as well as an important metabolic and immune organ. Intestinal cell metabolism is key to regulating intestinal homeostasis (Litvak et al., 2018). Among them, enteroendocrine cells secrete intestinal hormones that regulate metabolism by intervening in digestion and absorption, insulin secretion, and controlling appetite. GLP-1 is mainly produced by enteroendocrine L cells, and its release not only stimulates insulin secretion in pancreatic islets, but also indirectly inhibits gluconeogenesis in the liver. BBR can promote the secretion of GLP-1 in experimental diabetic rats, which may be related to the promotion of the proliferation of intestinal L cells in rats. BBR increases the messenger ribonucleic acid (mRNA) level of proglucagon and prohormone convertase 3 which control GLP-1 biosynthesis in intestinal endocrine cell line NCI-H716, thereby promoting GLP-1 biosynthesis. This process is associated with dependency protein kinase C pathway (Yu et al., 2010). In addition, BBR promotes intestinal GLP-1 secretion by targeting the activation of the bitter receptor taste receptor 2 member 38 expression in the intestinal mucosa (Yu et al., 2015). The activation of bitter receptors by BBR may be a potential target for the treatment of type 2 diabetes.

One of the main functions of intestinal cells is to absorb glucose from food and then transport it into the blood. This process is mediated by specific glucose transporters. BBR reduces intestinal glucose absorption by reducing glucose transport in intestinal epithelial cells-6. This process is mainly through inhibiting the insulin-like growth factor 1-phospholipase C (PLC)-β2-GLUT 2 signaling pathway, reducing localization of PLC-β2 in the membrane, and thereby reducing GLUT2 translocation (Zhang et al., 2021). The expression of sodium-dependent glucose cotransporter, another glucose transporter protein in intestinal epithelial cells, is also inhibited by BBR to some extent, but whether it can exert glucose-lowering effect at high glucose concentrations remains to be verified (Zhang et al., 2021) (Table 1)

**TABLE 1:** Summary of pharmacological and clinical studies on berberine regulating body metabolism.

Type of study	Models	Treatment, dose and duration	Positive controls	Negative controls	Findings	References
Clinical study	Newly diagnosed patients with type 2 diabetes	Berberine (0.6 g per 6 pills, twice daily before a meal) - oral, 12 weeks	Probiotics (4 g per 2 strips of powder, once daily at bedtime)	Placebo	Improving postprandial pTC and pLDLc levels	Wang et al. (2022)
Clinical study	Patients with hypercholesterolemia	Berberine (0.5 g twice daily) - oral, 3 months	N/A	Placebo	Significantly lowering serum levels of CHOL, TG, and LDL-C	Kong et al. (2004)
Clinical study	Patients with T2DM	Berberine (0.5 g twice daily) - oral, 3 months	Metformin (0.5 g 3 times daily)	N/A	Reduces HbA1c, FBG, PBG, TG, fasting plasma insulin, and HOMA of insulin resistance	Yin et al. (2008)
Clinical study	Healthy males receiving a 160-min hyperglycemic clamp experiment	Berberine (1 g) - oral, one time 1 h before the start of the clamp study	N/A	Placebo	Promotes insulin secretion under hyperglycemic state, which does not affect basal insulin secretion in humans	Zhao et al. (2021)
Clinical study	Subjects with polycystic ovary syndrome and insulin resistance	Berberine (3 × 500 mg daily)+ compound cyproterone acetate - oral, 3 months	Metformin (500 mg twice daily)	Placebo (one tablet twice daily)	Reduces waist circumference and waist-to-hip ratio, TC, TG, LDL-C, WHR, FPG, fasting insulin and HOMA-IR; elevates HDL-C and SHBG	Wei et al. (2012)
Clinical study	Patients who met DSM-IV-TR criteria for schizophrenia	Berberine (900 mg per day) - oral, 8 weeks	N/A	Placebo (900 mg daily)	Reduces body weight and BMI of patients	Qiu et al. (2022)
Animal	High-fat diet mice	Berberine (100 mg/kg) - oral, 10 weeks	Metformin (200 mg/kg/day)	N/A	Inhibits hepatic gluconeogenesis to lower glucose	Li, A. et al. (2018)
Animal	Ob/ob mice and high-fat diet mice	Berberine (300 mg/kg/day) - oral, 4 weeks	N/A	0.5 Carmellose sodium solution	Reduces the hepatic TG accumulation	Zhu et al. (2019)
Animal	Wildtype littermates and liver-specific SIRT1-deficient mice receiving a high-fat, high-sucrose diet	Berberine (5 mg/kg/day) - intraperitoneal injection, 5 weeks	N/A	PBS	Lowers hepatic steatosis and body weight	Sun et al. (2018)
Animal	ApoE−/− mice receiving a high-cholesterol diet	Berberine (10 mg/kg/d) - injection, 14 weeks	N/A	Vehicle	Reduces the size of atherosclerotic plaques and lowers cholesterol level	Yang et al. (2020)
Animal	Type 2 diabetic spontaneous gene mutant mice	Berberine (5 mg/kg) - injection, 3 weeks	N/A	Vehicle	Inhibits the proinflammatory response of adipose tissue in mice	Jeong et al. (2009)
Animal	T2DM mice induced by high-fat feeding plus low-dose intraperitoneal injection of STZ	Berberine (160 mg/kg) - oral, 4 weeks	N/A	0.9% NaCl	Reduces FBG levels and TG and TC contents; elevates HDL-C content; increases FINS levels; improves islet tissue area and insulin secretion	Lv et al. (2021)
Animal	Db/db spontaneous type 2 diabetes mice	Berberine (160 mg/kg, b.w.) - oral, 4 weeks	Huang-Gui Solid Dispersion (HGSD) (160 mg/kg, b.w.)	NS(10 mL/kg, b.w.)	Lowers FBG, RBG, and TG; improves the OGTT and ITT; increases the islet area; decreases inflammation and vacuolation	Li et al. (2019)
Animal	High-fat diet mice	Berberine (50 mg/kg) - oral, 4 weeks	N/A	N/A	Downregulates BECN1 expression in adipose tissue of mice and inhibits basal autophagy in adipose tissue of mice	Deng et al. (2014)
Animal	Obese db/db mice	Berberine (5 mg/kg/day) - injection, 4 weeks	N/A	Vehicle	Reduces body weight and free fatty acid levels; improves glucose tolerance; enhances energy expenditure and adaptive thermogenesis	Zhang et al. (2014)
Animal	High-fat diet mice	Berberine (25 and 100 mg/kg) - oral, 6 weeks	N/A	0.9% sterile saline	Reducing body weight and insulin resistance; Reducing plasma levels of T-CHO, TG, and LDL-C; Increasing the content of brown adipose tissue and adaptive thermogenesis	Xu, Y. et al. (2021)
Animal	High fat diet-induced obese mice	Berberine (75 and 150 mg/kg/day) - oral, 4 weeks	N/A	Vehicle	Improves glucose tolerance; attenuates collagen deposition; and reverses the upregulation of fibrosis associated genes	Wang, L. et al. (2018)
Animal	T2DM rats induced by fat feeding combined with STZ injection	Berberine (75 and 150 mg/kg/day) - oral, 4 weeks	N/A	N/A	Lowers FBG and fasting serum insulin and improves insulin sensitivity	Kong et al. (2009)
Animal	Diabetic rats induced by high-fat diet combined with STZ injection	Huang-Gui solid dispersion (a preparation of berberine coupled to sodium caprate) (25, 100 mg/kg) - oral, 4 weeks	N/A	0.5% Sodium carboxymethyl cellulose	Restores blood glucose and skeletal muscle glucocorticoid levels and improves insulin resistance	Meng et al. (2020)
Animal	Insulin-resistant rats induced by fructose	Berberine (100 mg/kg) - oral, 9 weeks	Metformin (300 mg/kg)	Vehicle	Improves body weight gain and abdominal circumference; reverses elevated blood glucose, AUC-ITT, HOMA-IR, and HOMA-ISI indices; improves dyslipidemia; alleviates inflammatory response; reduces pathological injury and muscle fiber fracture	Cheng, J. et al. (2023)
Animal	Rats with acute insulin resistance induced by systemic infusion of intralipid	Berberine (10 mg/kg) - intravenous, one time	N/A	Normal saline	Ameliorates intralipid-induced insulin resistance and alleviates mitochondrial swelling in skeletal muscle	Dong et al. (2021)
Animal	Db/db spontaneous T2DM mice	Berberine (5 mg/kg/d) - intraperitoneal injection, 4 weeks	N/A	Vehicle	Reduces serum FBG, insulin FFA, TG, and CHOL levels; decreases TG content in skeletal muscle; increases mitochondrial number in skeletal muscle	Yao, S. et al. (2020)
Animal	Western diet-induced atherosclerosis in ApoE−/− mice	Berberine (5 mg/kg/day) - oral, 12 weeks	Simvastatin (5 mg/kg)	Normal saline	Reduces serum levels of visfatin, lipid, interleukin-6, and tumor necrosis factor-α, the protein expression of visfatin, p-p38 MAPK, and JNK in mice aorta and the distribution of visfatin in the atherosclerotic lesions	Wan et al. (2018)
Animal	Diabetic rats induced by a high-fat diet combined with STZ	Berberine (100 mg/kg) - oral, 8 weeks	N/A	Normal saline	Reduces FBG and TG levels in diabetic rats and improves endothelium-dependent vasorelaxation impaired in aorta	Wang, C. et al. (2009)
Animal	Zucker diabetic fatty rats induced by a high-energy diet	Berberine (100 mg/kg/d) - oral, 3 weeks	N/A	Distilled water	Reduces food intake, FBG levels, insulin resistance, and plasma LPS level; enhances the number of goblet cells and villi length; improves the structure of the gut microbiota and the abundance of Gram-negative bacteria	Wang et al. (2021)
Animal	Zucker diabetic fatty (ZDF) rats	Berberine (100 mg/kg), oral, 69 days	Stachyose (200 mg/kg)	N/A	Reduces blood glucose; improves impaired glucose tolerance; and increases the abundance of beneficial *Akkermansiaceae*, while decreasing that of pathogenic *Enterobacteriaceae* in ZDF rats	Li et al. (2021)
Animal	Db/db spontaneous T2DM mice	Berberine (136.5 mg/kg) - oral, 11 weeks	Metformin (113.75 mg/kg)	Normal saline	Restores the intestinal SCFA content; reduces the level of serum LPS; relieves intestinal inflammation; repairs the intestinal barrier structure; modifies the gut microbiome; increases the number of SCFA-producing bacteria while decreasing opportunistic pathogens	Zhang, W. et al. (2019)
Animal	Normal rats with a glucose load	Berberine (60, 120 mg/kg) - oral, 5 weeks	N/A	Vehicle	Enhances GLP-1 secretion induced by glucose load, promoting proglucagon mRNA expression and L cell proliferation in intestine	Yu et al. (2010)
Animal	T2DM induced by STZ combined with a high fat diet	Berberine chloride (200 mg/kg/day) - oral, 6 weeks	N/A	0.9% saline solution	Alleviating hyperglycemia, improving Insulin sensitivity and reducing intestinal glucose absorption; Decreasing GLUT2 localization in brush border membrane of intestinal epithelial cells	Zhang et al. (2021)
Cell culture	FFA-induced steatosis HepG2 cells	Berberine (1, 5, and 25 μg/mL) - indicated	N/A	N/A	Reduces lipid accumulation and TC content	Shan et al. (2021)
Cell culture	Immortalized hepatocyte cell line MIHA	Berberine (10, 20, and 100 μM) - indicated	N/A	N/A	Increases LDLR mRNA levels in a dose-dependent manner	Li, C.H. et al. (2018)
Cell culture	Human monocytic cell line THP-1 induced by PMA and stimulated by ox-LDL	Berberine (5–50 µM) - indicated	N/A	N/A	Alleviates foam cell formation and decreases the cholesteryl esters/total cholesterol ratio in macrophages	Huang et al. (2012)
Cell culture	J774A.1 macrophages induced by ox-LDL	Berberine (6.25, 12.5, 25, and 50 μM) - indicated	N/A	Chloroquine (30 μM)	Reduces ox-LDL–induced inflammation	Fan et al. (2015)
Cell culture	THP-1-derived macrophages induced by PMA and ox-LDL	Berberine (5, 10, and 20 mg/L) - indicated	N/A	AMPK inhibitor (10 μM)	Inhibits ox-LDL-induced foam cell formation and cholesterol accumulation in macrophages	Chi et al. (2014)
Cell culture	LPS-induced NIT-1 and INS-1 β-cells	Berberine (1.25, 2.5, and 5 mM) - indicated	N/A	TLR4 inhibitor (1 mM)	Reduces LPS-induced insulin levels and β-cell apoptosis	Wang (2013)
Cell culture	Mouse primary preadipocytes	Berberine (5, 10, and 20 μmol/L) - indicated	N/A	DMSO	Suppresses the differentiation and proliferation of preadipocytes by downregulating Gal-3	Wang, C. et al. (2019)
Cell culture	Human NCI-H716 cells	Berberine (1, 10, and 100 µM) - indicated	PTC (1, 3, and 10 mM)	Anti-TAS2R38 antibody (1:200 and 1:400 diluted)	Stimulates GLP-1 secretion by activating the gut-expressed bitter taste receptors in a PLC-dependent manner	Yu et al. (2015)

In addition, some studies have shown that intestinal permeability is associated with certain metabolic diseases in humans (Gummesson et al., 2011; Teixeira et al., 2012). When intestinal mucosal barrier is damaged, bacterial components such as endotoxin and bacterial metabolites can enter blood circulation, triggering certain inflammatory signals in the body, and then leading to metabolic diseases. This process is associated with Toll-like receptor (TLR) transduction and proinflammatory cytokines expression (Massier et al., 2021). BBR can protect intestinal mucosal barrier injury and inhibit inflammatory response in early septic rats by targeting TLR signaling pathway (Li et al., 2015; Chen et al., 2018). In addition, BBR can also improve pro-inflammatory factor-induced tight junction injury in intestinal epithelial cell lines (Li et al., 2010), restore tight junction protein zone 1 levels in diabetes rats (Shan et al., 2013), and repair intestinal barrier. Some studies have supported the hypothesis that BBR alleviates diabetes and regulates intestinal function. It is believed that the hypoglycemic effect of BBR on type 2 diabetes rats may play a role partly by improving intestinal mucosal barrier, but further experiments are needed to confirm the link (Shan et al., 2013).

#### 2.1.2 BBR regulates the gut microbiota

The intestinal flora is regarded as a large metabolic ‘organ’ in the body that plays an important role in metabolic disorders. Intestinal microorganisms are involved in important physiological processes, such as nutrient absorption, substance metabolism, and immune defense, which affect host metabolism at many levels. Although the intestine is the main site of action of BBR, its oral bioavailability is poor and the mechanisms by which BBR regulates metabolic disorders are not yet fully understood. BBR has been shown to exert a metabolic protective effect by regulating the intestinal microbiota (Zhang et al., 2020; Wang et al., 2021); this effect can be attributed to an enhanced abundance of beneficial bacteria and a reduced abundance of harmful bacteria in the body. BBR also improves glucose metabolism in Zucker diabetic fatty rats by remodeling the intestinal flora, specifically reducing the abundance of pathogenic Desulfovibrionaceae and *Proteobacteria* (Li et al., 2021). In addition, BBR can improve obesity and insulin resistance in high-fat diet (HFD) rats, at least in part, by modulating the structure of the gut microbiota (Zhang et al., 2012). After BBR treatment, the improvement in insulin resistance in Zucker diabetic fatty rats was consistent with a decrease in the abundance of *Prevotella copri*, which is a bacterium that can directly affect host metabolism and induce obesity when combined with a high-fat diet. In addition, *Akkermansia* is a beneficial gut microbiota species that exerts a positive regulatory effect against obesity and metabolic disorders (Everard et al., 2013; Dao et al., 2016). BBR reportedly alleviates atherosclerosis in HFD mice by increasing the abundance of intestinal *Akkermansia muciniphila* species. BBR can impact certain short-chain fatty acid (SCFA) producers, which are known intestinal metabolites closely related to glucose and lipid metabolism (Wang et al., 2021). Treatment with BBR can increase the number of SCFA-producing bacteria in diabetes db/db mice, aid in restoring the intestinal SCFA content, and modify the structure of intestinal flora, thereby improving blood glucose levels and reducing diabetic complications (Zhang et al., 2019). Bile acid is an important metabolic signaling molecule closely related to lipid regulation (Fiorucci et al., 2010). BBR exerts a lipid-lowering role by activating the intestinal farnesoid X receptor signaling pathway and microbial metabolism of taurocholic acid in high-fat obese mice (Sun et al., 2017).

Furthermore, intestinal bacteria have been shown to metabolize oral BBR. It was found that the gut microbiota can use their nitroreductases to reduce BBR to an absorbable form known as dihydroberberine (dhBBR), which can then enter the bloodstream (Feng et al., 2015). In this process, the chemical structure and function of BBR is altered by intestinal microorganisms. This solves the problem of poor solubility and doubles BBR intestinal absorption rate, which is of great importance in drug research.

#### 2.1.3 BBR improves the disorder of glucose and lipid metabolism in hepatocytes

The liver is the primary source of endogenous glucose production: under basal conditions, about 80% of endogenous glucose production comes from hepatic glycogenolysis and gluconeogenesis (Klein et al., 2022). In lean individuals, both glycogenolysis and gluconeogenesis raise blood glucose, but in obese or type 2 diabetes patients, the contribution of gluconeogenesis is much higher. So suppressing hepatic gluconeogenesis is an effective way to control fasting blood glucose in diabetes (Gastaldelli et al., 2000). BBR has the therapeutic potential to lower blood glucose level by inhibiting hepatic gluconeogenesis. The MPC is composed of MPC1 and MPC2, and the input of pyruvate into the mitochondria is mediated by MPC. Once pyruvate enters the mitochondria, it is guided by the enzyme pyruvate carboxylase to undergo gluconeogenesis; the entry of pyruvate into the mitochondria through MPC is considered a pivotal step in hepatic gluconeogenesis. BBR inhibits SIRT-3 to protect the acetylation of MPC1 and disrupts the stability of the MPC1 protein through proteolysis, thereby limiting the supply of mitochondrial pyruvate for gluconeogenesis and reducing hepatic gluconeogenesis (Li et al., 2018). Glucagon physiologically induces hepatic gluconeogenesis. BBR reduces glucagon-stimulated cyclic adenosine 3'-5' monophosphate (cAMP) levels by activating phosphodiesterase, thereby downregulating the phosphorylation of the cAMP response element binding, and ultimately downregulating gluconeogenesis genes in liver cells, including phosphoenolpyruvate carboxykinase, glucose 6-phosphatase, and peroxisome proliferator-activated receptor-gamma coactivator 1-alpha (PGC-1α), thereby reducing hepatic gluconeogenesis (Zhong et al., 2020). Another study also showed that BBR can inhibit cAMP response element-binding protein phosphorylation and downregulate glucose 6-phosphatase and phosphoenolpyruvate carboxykinase, thereby inhibiting hepatic gluconeogenesis. This effect is related to the BBR-activated AKT-dependent mitogen-activated protein kinase (MAPK) pathway (Lu et al., 2023).

**FIGURE 1:**
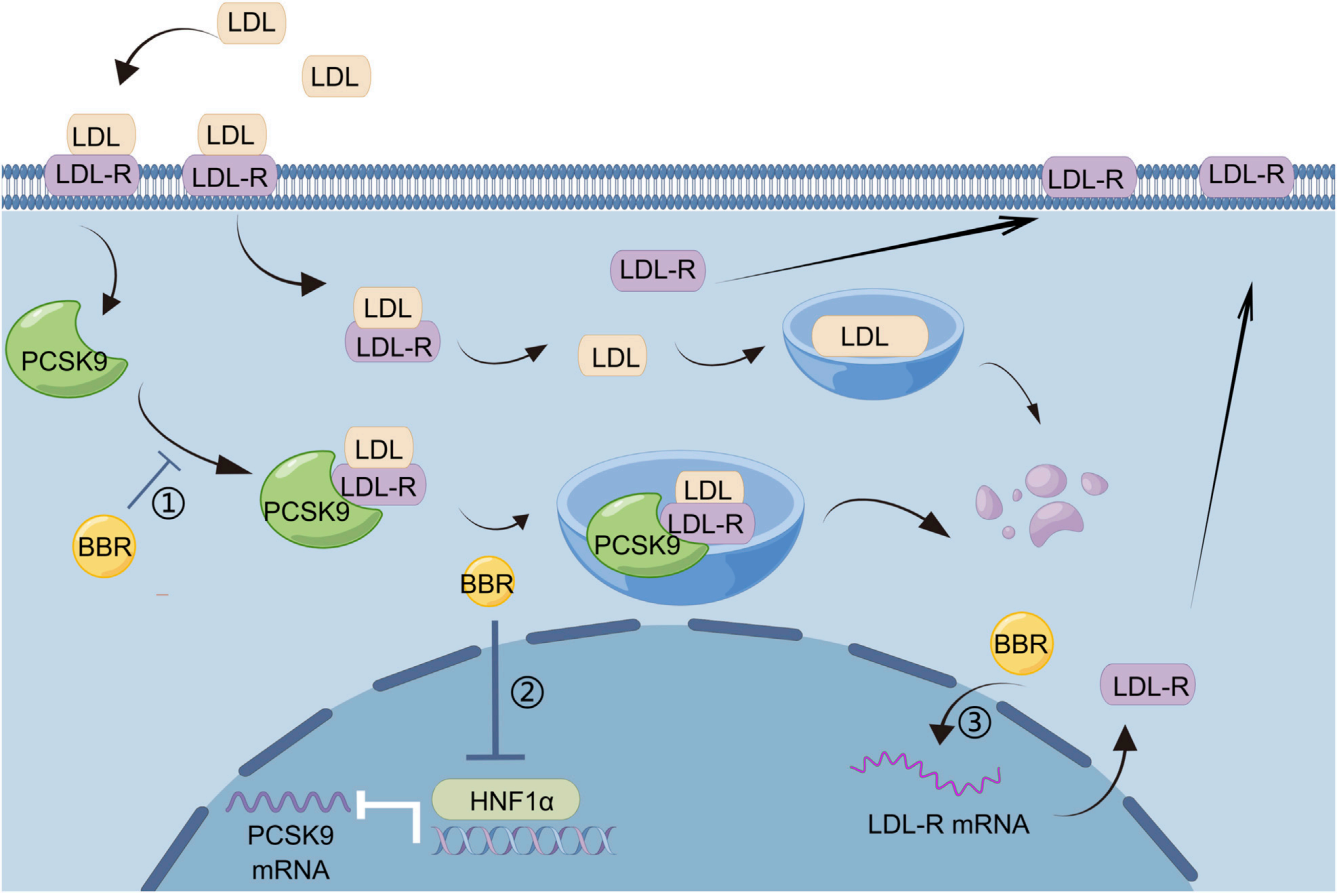
BBR regulates lipid metabolism by reducing PCSK9 levels: 1) BBR inhibits the formation of PCSK9–LDL-R–LDL complexes and reduces the degradation of LDL-R by lysosomes, 2) HNF1α mediates the transcription of PCSK9, and BBR can reduce PCSK9 mRNA and protein levels by decreasing the content of HNF1α protein in liver, 3) BBR can increase the uptake of LDL-C by enhancing the stability of LDL-R mRNA and increasing LDL-R density in hepatocytes.

The liver is the main site for lipid metabolism. Digestion, absorption, transport, catabolism, and anabolism of lipids are closely linked to liver metabolism. Excessive fat intake leads to excess fat accumulation in liver cells in the form of lipid droplets, and intrahepatic fat accumulation may lead to hepatic fat metabolism disorders. Once liver fat metabolism disorder occurs, it not only causes liver disease but may also aggravate metabolic disorders in the body. BBR has good efficacy and safety in the treatment of patients with hyperlipidemia without the adverse reactions seen for statins (Wang et al., 2022), and is a promising lipid-lowering drug. BBR regulates lipid metabolism by acting on hepatocytes, and proprotein convertase subtilisin/kexin type 9 (PCSK9) is an important target. PCSK9 is a serine protease that is mainly synthesized and secreted by the liver. It mainly binds to LDL receptor (LDL-R) to promote its degradation, thereby reducing the clearance of LDL cholesterol (LDL-C). LDL-C binds to LDL-R on the membrane surface of hepatocytes and enters hepatocytes *via* endocytosis, and a decrease in intracellular pH dissociates LDL-C from LDL-R. After dissociation, LDL-C is degraded in lysosomes, whereas LDL-R returns to the hepatocyte membrane surface and continues to bind to the remaining LDL-C. PCSK9 binds to LDLR to form PCSK9–LDL-R-LDL complexes, which together enter lysosomes for degradation, leading to a decrease in LDL-R on the surface of hepatocytes and a corresponding decrease in the degradation of LDL-C (Liu et al., 2022). BBR, a natural cholesterol-lowering metabolite, has been found to be a promising PCSK9 inhibitor (Momtazi et al., 2017). It has been demonstrated that PCSK9 is upregulated by hepatic nuclear factor 1 alpha (HNF1α) and sterol regulatory element-binding protein (SREBP)-2 transcription factors, and BBR inhibits HNF1α-mediated PCSK9 transcription by reducing hepatic HNF1α protein content without affecting its mRNA levels (Dong et al., 2015), thereby reducing PCSK9 mRNA and protein levels (Cameron et al., 2008). BBR also increased LDL-C uptake by enhancing the stability of LDL-R mRNA in hepatocytes and increasing LDL-R density in hepatocytes (Kong et al., 2004). In animal models of dyslipidemia, BBR had no effect on the mRNA or protein expression of SREBP-2 when it reduced PCSK9 expression (Dong et al., 2015; Liu, D.-l. et al., 2015). Many current lipid-lowering drugs upregulate LDL-R by stimulating SREBP-2, leading to PCSK9-mediated drug resistance in dyslipidemia, which can be regarded as a useful adjunct to statin therapy (Figure 1).

Hepatic steatosis is a common consequence of metabolic or toxic stress, and long-term hepatocyte degeneration can lead to hepatocyte damage. This in turn can lead to liver fibrosis and cirrhosis (Koyama and Brenner, 2017); therefore, early intervention is very important. The liver is a central metabolic organ that regulates lipid homeostasis, and reducing lipid production can effectively improve hepatic steatosis. BBR reduces liver triglyceride (TG) synthesis and alleviates hepatic steatosis by activating the AMPK-SREBP-1c-stearoyl-coenzyme A desaturase 1 (SCD1) pathway. Specifically, increased phosphorylation of AMPK promotes the phosphorylation of SREBP-1c and inhibits its cleavage and nuclear translocation. The binding of SREBP-1c to the sterol regulatory element motif on the promoter of SCD1 is reduced, resulting in the decreased expression of SCD1, thus alleviating hepatocyte steatosis (Zhu et al., 2019). BBR may reduce cholesterol synthesis and alleviate non-alcoholic hepatic steatosis by downregulating the SIRT1-forkhead box O (FOXO)1-SREBP2 signaling pathway in HepG2 cells. This is evidenced by SIRT1 and FOXO1 inhibitors blocking the effects of BBR treatment (Shan et al., 2021). Lipoautophagy regulates intracellular lipid homeostasis in response to various nutritional signals, and impaired lipoautophagy leads to excessive lipid accumulation in the liver (Liu and Czaja, 2013). BBR alleviates hepatic steatosis and controls energy balance in mice by inducing autophagy and fibroblast growth factor (FGF)-21, where SIRT1 is critical to this process (Sun et al., 2018). BBR stimulates SIRT1 deacetylation and induces autophagy in hepatocytes in an autophagy protein 5-dependent manner. In addition, BBR induced the expression of micro-RNA (miR)-373 in non-tumor hepatic MIHA cells. Upregulation of miR-373 depletes the levels of mRNA of target gene serine/threonine-protein kinase 1 (AKT1), and depletion of AKT1 results in decreased AKT1-mammalian target of rapamycin (mTOR) pathway activity. This is followed by inhibition of phosphorylated S6 kinase levels in hepatocytes, thereby attenuating S6K-mediated hepatic steatosis (Li, C.H. et al., 2018).

### 2.2 Circulatory system

Metabolic intermediates such as sugars, fats, and proteins are transported through the circulatory system to ensure normal cellular metabolism. When important cells in the circulatory system are damaged, it can lead to changes in the composition and properties of substances in the blood, resulting in the accumulation of local metabolites or inflammatory products, causing circulation and metabolic disorders. Among them, endothelial cells and macrophages have a significant influence.

#### 2.2.1 BBR improves endothelial function and vascular inflammation

Endothelial cells have important endocrine functions, regulating vascular function through the secretion of vasoactive substances. Abnormal endothelial cell metabolism can lead to various metabolic diseases, which are related to endothelial dysfunction, inflammation, and oxidative stress (Deanfield et al., 2007). When endothelial cells are damaged by pathological stimuli, other cells in the blood (such as smooth muscle cells) aggregate and form a fibrin network with fibrin to repair the damage to endothelial cells. However, when the vascular endothelium is severely damaged, endothelial cells will secrete a large number of growth factors and active substances, attract too many monocytes to adhere to them, and migrate to deposit in the intima of blood vessels. Monocytes differentiate into macrophages and then absorb oxidatively modified LDLs in the blood, forming foam cells, which become potential risk factors for atherosclerosis (Rui et al., 2021). In response to this, BBR inhibits certain cell factors or proteins that stimulate endothelial cell damage. Adiponectin, with its pro-inflammatory effect, induction of cholesterol accumulation, and regulation of scavenger receptors (SR)-A and CD36 expression, has a harmful effect in atherosclerosis. BBR improves mouse endothelial function and alleviates atherosclerosis by inhibiting p38 MAPK and c-Jun N-terminal kinase (JNK) signaling pathways and downregulating adiponectin expression (Wan et al., 2018). Another study showed that BBR exhibited strong antioxidant capacity against LDL oxidation and had a protective effect against oxidized (ox) LDL-induced endothelial cell toxicity. In this process, BBR primarily inhibited oxLDL-induced reactive oxygen species (ROS) production, mitochondrial membrane damage, and cell apoptosis, including the inhibition of cytochrome C release and cysteine-aspartic protease-3 activation (Hsieh et al., 2007).

Vascular inflammation is one of the main causes of vascular remodeling (Hansson, 2005), especially high oxidative stress and low nitric oxide utilization. BBR improves the bioavailability of nitric oxide by reducing the protein expression of nicotinamide adenine dinucleotide phosphate oxidase (NOX) 4 in endothelial cells, thereby downregulating the expression of NOX. This is the main source of ROS production by endothelial cells, and ultimately improves endothelial function of diabetes rats (Wang et al., 2009). Endothelial nitric oxide synthase (eNOS) acts as a key enzyme for nitric oxide production in endothelial cells and its upstream kinases include AMPK, Akt, and protein kinase A (Mount et al., 2007). Among them, AMPK plays a central role in BBR-induced endothelial nitric oxide production and vasodilation, which promotes the binding of eNOS to heat shock protein 90 and thus maximizes eNOS activation and NO release, protecting against endothelial cell damage caused by high glucose. When AMPK is inhibited, BBR-mediated eNOS phosphorylation, NO production, and endothelium-dependent vasodilation are all eliminated (Wang et al., 2009). Endoplasmic reticulum (ER) stress in endothelial cells exacerbates oxidative stress. BBR may attenuate endothelial-dependent vasoconstriction by activating AMPK phosphorylation in the carotid arteries of spontaneously hypertensive rats, thereby inhibiting ER stress and protecting endothelial function. In this process, BBR inhibits the phosphorylation of eukaryotic translation initiation factor 2A and attenuates the expression of activating transcription factor-3, activating transcription factor-6, and X-box binding protein-1 (Liu et al., 2015). These three proteins mediate ER stress and are involved in complex downstream signaling pathways (Yang et al., 2010).

In addition, BBR participates in the regulation of lipid metabolism, thereby improving endothelial function. Treatment with BBR reversed and upregulated paraoxonase-1 (PON1) and apolipoprotein A1 (APOA1) expression and downregulated glycerol-3-phosphate dehydrogenase 2 (GPD2) expression in apolipoprotein E-deficient mice (Tan et al., 2020). These three proteins are strongly associated with atherosclerosis and lipid metabolism. As an antioxidant, paraoxonase-1 can hydrolyze lipid hydroperoxides and prevent oxidation of LDL (Aviram et al., 2005). GPD2 can regulate the transition of the inflammatory response and inhibit the oxidative metabolic response (Langston et al., 2019). APOA1 is the main protein component of HDL in the plasma and promotes the outflow of cholesterol from tissues. Studies have shown that regulating APOA1 transcription and increasing APOA1 levels in mice can reduce atherosclerosis (Guo et al., 2015). Thus, APOA1 may be a potential target for BBR to exert endothelial protection.

### 2.3 Immune system

#### 2.3.1 BBR inhibits the formation of foam cells derived from macrophages and stabilizes plaques

Atherosclerosis is the most important pathological process in the development of cardiovascular diseases. Dysregulation of lipid metabolism and abnormal inflammatory responses are considered major risk factors for atherosclerosis. BBR is a candidate for the treatment of atherosclerosis due to its pleiotropic antidyslipidemic and anti-inflammatory effects. Macrophage-derived foam cells play a key role in both the early and late stages of atherosclerosis (Chinetti-Gbaguidi et al., 2015); BBR can reduce foam cell formation. The process of macrophages taking up oxLDL to become foam cells depends on SRs, such as SR-A type I, cluster of differentiation (CD)36, and lectin-like oxLDL receptor (LOX)-1. The adenosine triphosphate (ATP)-binding cassette transporter G1 (ABCG1) and SR-B type I in macrophages participate in reverse cholesterol transport, and the upregulation of these proteins can weaken the formation of foam cells. BBR can reduce oxLDL uptake by inhibiting scavenger receptors CD36 and LOX-1, and increase cholesterol efflux by inhibiting the expression of adipocyte enhancer-binding protein 1 in macrophages (Huang et al., 2012). Another study showed that BBR can inhibit the influence of oxLDL on the foam cell formation from macrophages by inhibiting LOX-1 and upregulating SR-B type I expression (Guan et al., 2010). ABCG1 plays an important role in cholesterol efflux from foam cells. ABCG1 can directly mediate the efflux of cholesterol from the cells (Rosenson et al., 2016), and BBR can upregulate ABCG1 through nuclear factor, erythroid 2-related factor 2 (Nrf2)/HO-1 signaling in human macrophages, thereby regulating lipid homeostasis and inhibiting macrophage foam cell formation (Yang et al., 2020). BBR inhibits foam cell formation by activating the AMPK-SIRT1-proliferator-activated receptor γ (PPAR-γ) pathway and reducing oxLDL uptake. Compared with atorvastatin alone, the use of BBR in combination with atorvastatin can more effectively prevent the process of atherosclerosis (Chi et al., 2014).

Macrophages are prominent inflammatory cells in atherosclerotic lesions and produce a series of inflammatory factors, such as interleukin (IL)-1β, tumor necrosis factor (TNF)-α, IL-6, IL-8, monocyte chemoattractant protein-1 (MCP-1), triggering inflammation-induced atherosclerosis. BBR reduces inflammatory responses by activating AMPK to downregulate the secretion of various inflammatory factors in macrophages (Jeong et al., 2009). Additionally, BBR inhibits the secretion of inflammatory factors in oxLDL-induced J774A.1 cells by inducing autophagy, a process related to the activation of the AMPK/mTOR signaling pathway (Fan et al., 2015). In the treatment of atherosclerosis, it is of great significance to prevent atherosclerotic plaque rupture, which can cause thrombosis, leading to myocardial infarction, stroke, and death. Plaques with large lipid cores and covered by thin fibrous caps have a higher risk of rupture (Schaar et al., 2004). Macrophages and foam cells produce matrix metalloproteinases and extracellular matrix metalloproteinase inducers, which digest extracellular matrix proteins and weaken the fibrous cap, thereby reducing the stability of atherosclerotic plaques. BBR activates the p38 pathway to reduce the expression of matrix metalloproteinase-9 and extracellular matrix metalloproteinase inducer in macrophages to effectively stabilize the plaque (Huang et al., 2011). Another study also demonstrated the role of BBR in stabilizing plaques. In oxLDL-induced macrophages, BBR significantly increased miR-150-5p, reduced P2X7R expression, and downregulated the expression of matrix metallopeptidase 9 and EMMPRIN (Lu et al., 2021). In conclusion, BBR has the potential to prevent or treat atherosclerotic plaque formation and progression.

### 2.4 Endocrine system

The endocrine system is closely related to metabolism, with endocrine cells synthesizing and secreting various hormones to distribute and transfer information between cells. Insulin secreted by islet cells binds to specific receptors on other target cells, producing a regulatory effect on the cells, such as adipocytes. Insulin resistance is often the core mechanism of dysregulated sugar and fat metabolism, and adipocyte metabolism is an important determinant of systemic insulin sensitivity.

#### 2.4.1 BBR regulates insulin secretion and protects islet cells

Type II diabetes mellitus is a growing epidemic characterized by insulin resistance, relative insulin deficiency, and islet β-cell failure. BBR can reduce HbA1c and fasting glucose levels in type II diabetes patients, and its efficacy and safety have been demonstrated in clinical trials (Yin et al., 2008). As an effective oral hypoglycemic agent, BBR can regulate glucose metabolism *in vitro* and *in vivo*, and its hypoglycemic effect is closely related to the regulation of insulin secretion and improvement of islet β cell function.

BBR, the main active metabolite of *Rhizoma coptifolia rhizoma*, has been used in traditional Chinese medicine to treat diabetes for thousands of years. The hypoglycemic effect of BBR has been verified in a variety of animal experiments and human clinical trials, and this may be achieved by stimulating islet β-cells to secrete insulin. SIRT1 is involved in the regulation of various physiological activities of islet β-cells, and the activation of SIRT1 has a beneficial effect on β cell function (Kitada et al., 2019). BBR enhanced insulin secretion through the miR-204/SIRT1 signaling pathway. MiR-204 reduces insulin production and SIRT1 expression in mouse INsulinoma 6 cells, but BBR blocks this effect, instead promoting insulin secretion in islet tissues of diabetic mice (Lv et al., 2021). Insulin secretion is also regulated by ATP-sensitive K^+^ channels, which have been used as targets of insulin secretagogues. The potassium voltage-gated channel subfamily H member 6 (KCNH6) is a voltage-dependent K^+^ channel. BBR directly binds to the KCNH6 channel and accelerates channel closure, blocking outward K_v_ channel currents and prolonging the duration of action potential in islet β-cells. This causes Ca^2+^ influx into cells via voltage-gated calcium channels, and intracellular Ca^2+^ concentration triggers increased insulin secretion. Clinical trials have shown that BBR significantly promotes insulin secretion in the hyperglycemic state without affecting human basal insulin secretion; therefore, some researchers believe that BBR is an insulin secretagogue, and is different from other insulin secretagogues, because BBR only effectively reduces blood glucose levels under hyperglycemic conditions without causing hypoglycemia targeting KCNH6 channels (Zhao et al., 2021). However, it has been reported that BBR can reduce insulin secretion and inhibit the insulin gene promoter by activating AMPK in mice. This may be due to AMPK activation in the muscle and liver improving insulin resistance, reducing insulin demand, and reducing the synthetic burden on β-cells (Shen et al., 2012).

**FIGURE 2:**
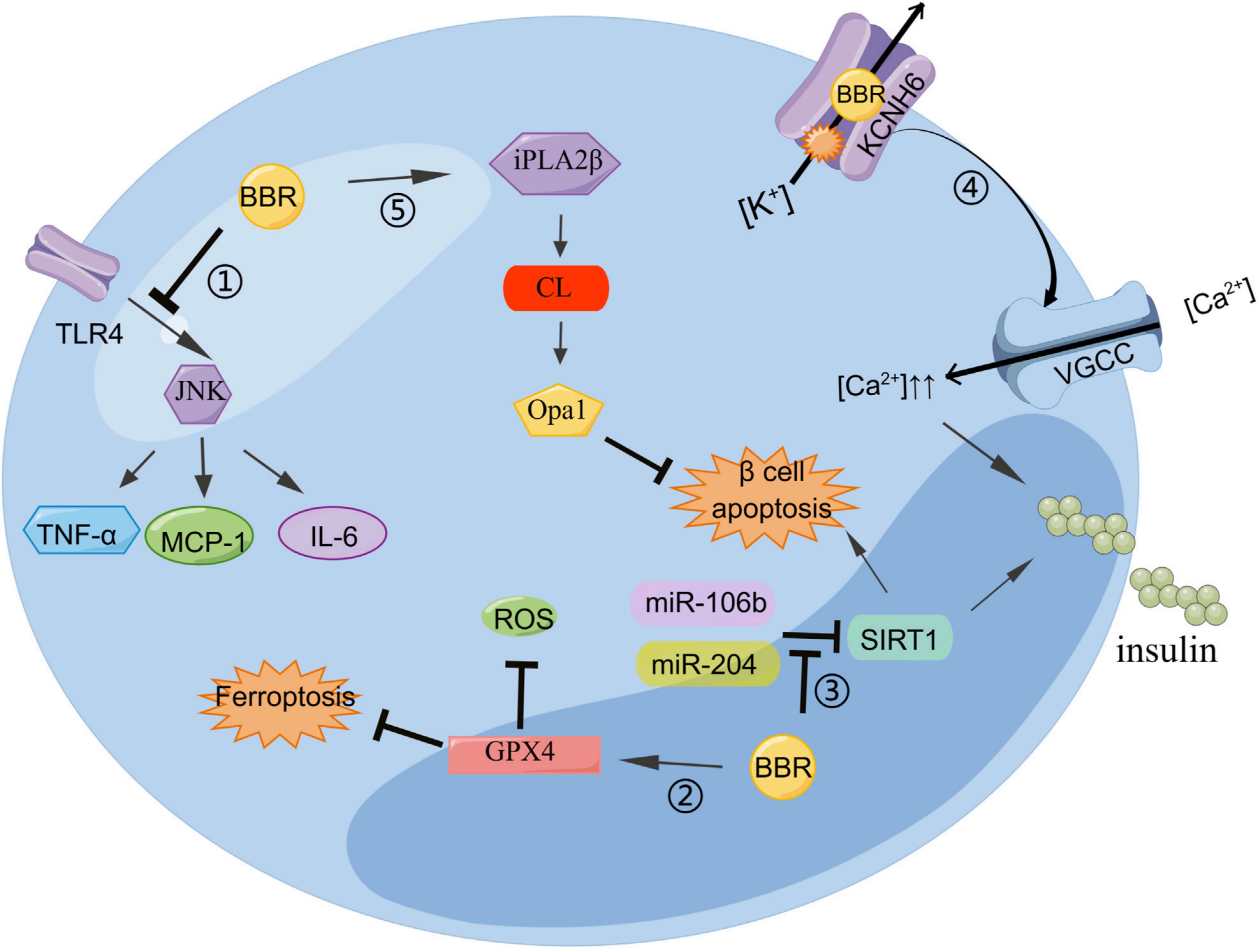
BBR can regulate insulin secretion and improve islet β cell function: 1) BBR inhibits the TLR4/JNK pathway and reduces MCP-6, IL-1, and TNF-α to alleviate cellular inflammation. 2) BBR stimulates GPX4 expression to reduce Fe^2+^ and ROS, thereby inhibiting ferroptosis in islet β-cells. 3) miR-204 decreases insulin production and SIRT1 expression in MIN6 cells, which was improved by BBR treatment. BBR inhibited miR-106b expression, upregulated SIRT1 expression, and weakened cell apoptosis. 4) BBR binds directly to KCNH6 channels, blocking outward Kv channel current and increasing intracellular Ca^2+^ concentration, thereby triggering more insulin secretion. 5) BBR attenuates β cell apoptosis by enhancing the iPLA2β/CL/Opa1 signaling pathway.

BBR not only stimulates the secretion of insulin but also protects islet β-cells. The inflammatory response can change the normal structure of β-cells, induce insulin resistance, and reduce insulin secretion. Reducing the inflammatory response improves β-cell function. BBR can inhibit the lipopolysaccharide-induced inflammatory response in pancreatic β-cells by interfering with the TLR 4/JNK/nuclear factor kappa-light-chain-enhancer of activated B cells (NF-κB) pathway. The lipopolysaccharide can increase the levels of inflammatory cytokines such as MCP-1, IL-6, and TNF-α. BBR may alleviate inflammatory injury by blocking early intracellular events in TLR4 signaling (Wang, 2013). BBR can inhibit β-cell apoptosis by upregulating calcium-independent phospholipase A2β (iPLA_2_β), cardiolipin (CL), and optic atrophy 1 (Opa1). The activation of iPLA_2_β is necessary for islet β-cells to secrete glucose to stimulate insulin. In iPLA_2_β silenced cells, the insulinogenic effect of BBR was weakened. In addition, CL/Opa1 expression was upregulated and decreased (Li et al., 2019). The occurrence and development of oxidative stress can lead to cardiovascular disease in diabetes. BBR inhibits the expression of miR-106b and upregulates SIRT1 to alleviate oxidative stress in islets of diabetic mice (Chen and Yang, 2017). Thus, BBR may reduce the incidence of diabetic cardiovascular diseases. Ferroptosis, a type of non-apoptotic cell death, has been linked to the pathogenesis of type I diabetes. BBR stimulation of GPx4 expression reduces Fe^2+^ and ROS, thereby inhibiting ferroptosis in islet β-cells. Its function is similar to that of the ferroptosis inhibitor ferrostatin-1 (Bao et al., 2022), which provides a new approach for improving the function of islet β-cells (Figure 2).

#### 2.4.2 BBR action in fat cells

Obesity is defined as the accumulation of excess adipose tissue caused by various metabolic disorders. The development of adipocyte dysfunction is closely related to the development of obesity and insulin resistance. BBR can reduce body weight in overweight individuals and improve metabolic disorders in polycystic ovary syndrome, and its main effect may be related to changes in body composition in patients with obesity and dyslipidemia (Wei et al., 2012; Qiu et al., 2022). BBR has shown significant anti-obesity and functional improvement effects on adipocytes both *in vivo* and *in vitro*, partly owing to its action in adipocytes.

##### 2.4.2.1 Anti-obesity activity of BBR

Obesity is a most common result of chronic energy metabolism imbalance and is considered an important risk factor for the development of type II diabetes and other metabolic diseases. Accumulation of fat is the direct cause of obesity, and many studies have shown that BBR can directly act on adipose tissue to achieve anti-obesity effects. Brown adipocytes are potential therapeutic targets for obesity-related metabolic diseases and stimulate FGF21 expression by regulating the molecular clock brain and muscle Arnt-like 1 in brown adipocytes. FGF21 secretion may act in a paracrine manner to promote thermogenic gene expression in brown adipocytes, thus alleviating diet-induced obesity (Hirai et al., 2019). Adipocyte differentiation and proliferation are regulated by a series of factors or molecules, among which galectin-3 (Gal-3) stimulates adipocyte differentiation and proliferation and is positively correlated with obesity. BBR downregulates Gal-3 by affecting its transcription, thereby inhibiting the differentiation and proliferation of preadipocytes isolated from epididymal white adipose tissue (WAT) used for fat storage, which contributes to its anti-obesity effect. Along with the downregulation of Gal-3 in differentiated adipocytes, the expression levels of adipogenic genes, such as PPARγ, were also reduced by BBR (Wang et al., 2019). The accumulation of TG in adipocytes contributes to obesity, therefore, reducing TG content in adipocytes is important to combat obesity. Adipose TG lipase is the rate-limiting enzyme that regulates the lipolysis of TG in adipocytes and mainly catalyzes the hydrolysis of TG to diacylglycerol. BBR directly increased the expression of adipose TG lipase in 3T3-L1 adipocytes and decreases TG levels in adipocytes (Jiang et al., 2016). In adipose tissue, increased autophagy may contribute to the pathogenesis of obesity (Kovsan et al., 2011) and plays an important role in regulating cellular metabolism and energy homeostasis. Beclin 1 (BECN1) is an essential protein for autophagy in differentiated 3T3-L1 adipocytes. BBR inhibits basal autophagy in mature adipocytes of fed mice by downregulating BECN1 expression by reducing the stability of BECN1 mRNA through the microRNA 30 family (Deng et al., 2014).

##### 2.4.2.2 BBR promotes browning of white adipose tissue

Brown adipocytes play a key role in the burning of glucose and lipids to maintain brown adipose tissue thermogenesis. In response to cold stimulation or β3-adrenergic receptor agonists, white adipocytes turn into brown-like adipocytes, a process known as the browning of white adipose tissue. Beige adipocytes are white or brown adipocytes. Uncoupling protein-1 (UCP1) is a hallmark of brown adipocytes. UCP1 can uncouple cellular respiration and ATP synthesis, contribute to heat production, and prevent obesity, and an increase in UCP1 content in WAT leads to a significant increase in whole-body energy expenditure. BBR can attract PGC-1α to UCP1 promoters by activating AMPK and inducing the expression of thermogenic genes, such as UCP1. Therefore, BBR can significantly induce the development of brown-like adipocytes in the groin (Zhang et al., 2014). PPARγ also promotes the expression of UCP-1 specific gene in brown adipocytes, inhibits the expression of specific genes in white adipocytes, and induces a brown-like phenotype in WAT. Selective deacetylation of PPARγ may be a regulatory switch in WAT conversion to brown adipose tissue. BBR activates the AMPK/SIRT1 axis to increase the level of PPARγ deacetylation, thereby regulating browning of adipose tissue and increasing the expression of the thermogenic protein UCP-1 (Xu et al., 2021). BBR can act as a selective PPARγ activator to promote adipose tissue remodeling and thermogenesis. BBR promotes white adipose tissue browning not only by regulating the expression of UCP-1 but also by affecting the genes related to brown adipogenesis. BBR treatment reduces the activity of the *c13orf25* promoter, which drives the transcription of miR-92a. Reduction in miR-92a leads to an increase in the RNA-binding motif protein 4a protein, which activates gene expression and splicing networks involved in brown adipogenesis (Lin et al., 2019). However, individual studies have shown that BBR selectively promotes brown adipogenesis through AMPK activation but has no significant effect on beige adipogenesis (Han et al., 2020). Further studies are needed to investigate the mechanism of BBR-promoted browning of white adipose tissue.

##### 2.4.2.3 BBR improves insulin resistance

Insulin resistance is a feature of metabolic diseases, such as obesity and type II diabetes. Insulin resistance is associated with the adipose tissue and chronic low-grade inflammation in adipose tissue is essential for its development. BBR upregulates the expression of SIRT1 in 3T3L-1 adipocytes, thereby inhibiting obesity-related inflammatory responses and improving local and systemic insulin resistance (Shan et al., 2020). BBR may also exert anti-inflammatory effects and ameliorate insulin resistance by inhibiting the phosphorylation of inhibitor kappa B kinase β Ser (Yi et al., 2008). In addition to low-grade inflammation in adipose tissue, adipose tissue fibrosis also contributes to obesity-related insulin resistance. BBR can significantly improve glucose tolerance in HFD-fed mice by inhibiting the abnormal deposition of a extracellular matrix in white adipose tissue and inhibiting the upregulation of fibrosis genes related to transforming growth factor-β1 signaling (Wang et al., 2018). BBR also regulates the cytokines secreted by adipocytes, such as retinol-binding protein 4. Retinol binding protein 4 is negatively correlated with the expression of GLUT4 during insulin resistance and BBR can significantly reduce the serum retinol binding protein 4 levels and upregulate the expression of GLUT4 in T2DM rats (Zhang et al., 2008). In addition, methylation of hypoxia-inducible factor-3α has previously been shown to be highly correlated with insulin resistance in patients with gestational diabetes and is considered a promising therapeutic target. BBR has been shown to be effective in improving insulin sensitivity in adipocyte models of insulin resistance, which may depend on reduced methylation and increased expression of hypoxia-inducible factor-3α (Wang et al., 2019).

### 2.5 Motor system

The skeletal muscle is the main organ of the movement system. The normal life activities of skeletal muscle cells also require the supply of various substances and energy. Skeletal muscle cells are the main target cells for human glucose uptake, metabolism, and utilization mediated by insulin. BBR can enhance the sensitivity of skeletal muscle to insulin, increase the uptake and metabolism of glucose by skeletal muscle, thereby regulating glucose metabolism. The Insulin Receptor (InsR) protein is a complete cell membrane glycoprotein and is crucial for the binding of insulin to target cells. Insulin-resistant individuals lack or reduce the expression of InsR in peripheral tissues. In *in vitro* models, BBR increases the expression of InsR in L6 skeletal muscle cells and glucose consumption by 97% in L6 skeletal muscle cells in the presence of insulin (Kong et al., 2009). Insulin signal transduction is defective in metabolic diseases. Under insulin resistance, BBR treatment improves insulin-mediated glucose transport in muscle tubes and GLUT4 translocation, promoting glucose uptake. In L6myc cells, BBR improves insulin-induced tyrosine phosphorylation of insulin receptor substrate 1 and the recruitment of p85 to insulin receptor substrate 1. These results show that BBR improves insulin signal transduction in muscle cells and may overcome insulin resistance by regulating key molecules in the insulin signaling pathway (Liu et al., 2010). Excessive glucocorticoids can induce insulin resistance and obesity through intracellular glucocorticoid receptors. Excessive glucocorticoid receptors in skeletal muscle may play an important role in the occurrence and development of type 2 diabetes. *In vivo* and *in vitro* experiments have shown that BBR preparations with high bioavailability can reduce glucocorticoid receptor-mediated skeletal muscle insulin resistance. This mechanism is related to its reduction of the binding of glucocorticoid receptors to phosphatidylinositol-3-kinase (Meng et al., 2020). Oxidative stress is closely related to the occurrence and development of insulin resistance. BBR has a protective effect on fructose-induced rat insulin resistance. BBR treatment significantly upregulates Nrf2 and HO-1 protein expression in rat skeletal muscle, and can inhibit malondialdehyde levels, alleviate oxidative stress by increasing superoxide dismutase, catalase and glutathione levels, and at least partially improve insulin resistance through the Nrf2/HO-1 pathway (Cheng et al., 2023). Although the pathogenesis of insulin resistance is still unclear, elevated intralipid can induce insulin resistance (IR). The expression of cyclophilin D (CypD) is closely related to IR. Compared to high-fat diet mice, CypD-deficient mice have significantly reduced high-fat diet-induced IR (Tavecchio et al., 2015). BBR relieves intralipid-induced insulin resistance in skeletal muscle by inhibiting the expression of CypD, without affecting blood glucose (Dong et al., 2021). Therefore, BBR clinical intervention for IR before the onset of diabetes may be a very promising strategy for diabetes prevention.

**FIGURE 3:**
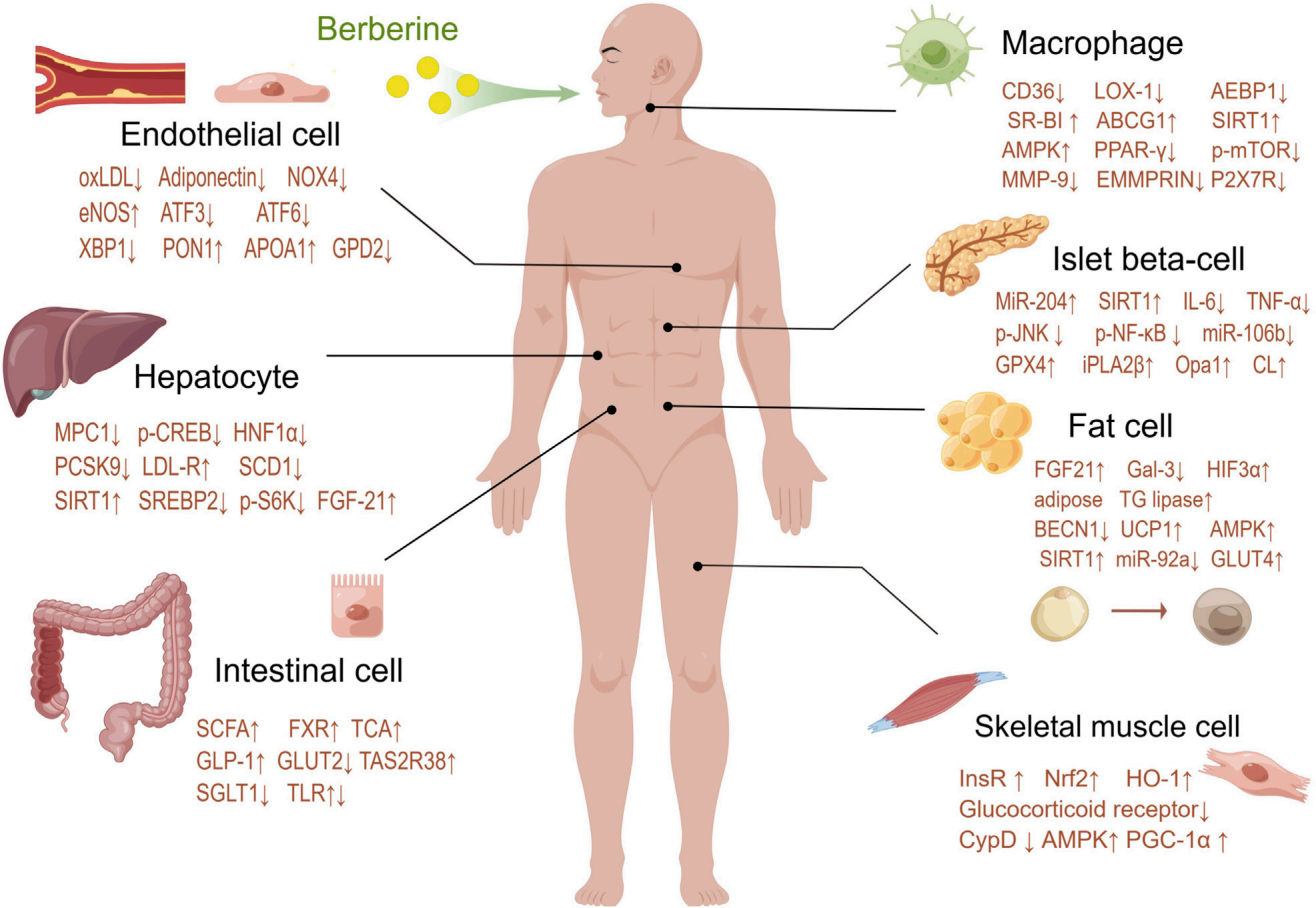
Berberine modulates targets of various systems and cells in the human body.

Intracellular lipid accumulation in muscle cells may exacerbate metabolic disorders, higher intramuscular lipid content is observed in insulin-resistant subjects (Kim et al., 2000). BBR is thought to be able to regulate skeletal muscle lipid metabolism and prevent lipid deposition in skeletal muscle. BBR can improve the quantity and function of skeletal muscle mitochondria, which may be the target of BBR to improve metabolic disorders in patients with type 2 diabetes. BBR promotes the expression of the key transcriptional coactivator PGC-1α through the AMPK pathway. PGC-1α has been proven to be a major regulator of mitochondrial biosynthesis and function, able to increase the number and function of mitochondria, and prevent ectopic lipid deposition in skeletal muscle (Yao et al., 2020) (Figure 3).

### 2.6 Other metabolism-related diseases

In addition to metabolic mechanisms associated with various systems and those typically observed in cells, BBR exerts metabolic regulatory effects on specific cell types associated with other diseases. Fibroblast-like synoviocytes (FLS) are the main cell type in the synovial membrane of joints, and in rheumatoid arthritis, these cells cause joint destruction by invading cartilage. In the diseased state, FLS proliferate excessively and become abnormally activated, interacting with migrating immune cells to secrete large amounts of inflammatory factors. Under the induction of IL-21 and adjuvants, FLS undergo uncontrolled proliferation (Noack and Miossec, 2017). BBR was found to suppress this excessive proliferation by increasing BAX expression and decreasing B cell lymphoma-2 (Bcl-2) levels (Dinesh and Rasool, 2019). During autophagy, which is an intracellular catabolic mechanism, BBR reportedly inhibits LC3-I/II autophagy factors and activates CHOP to suppress AA-FLS autophagy response (Dinesh and Rasool, 2019). Moreover, BBR may exert anti-arthritic effects by regulating the balance between Treg and Th17 cells (Wang et al., 2017). BBR suppressed the proliferation and differentiation of Th17 cells by downregulating RORγ-t and CD196 and induced Foxp3 expression by activating the aryl hydrocarbon receptor, thereby promoting Treg cell differentiation and ultimately regulating the imbalance of Th17/Treg (Dinesh and Rasool, 2019). Furthermore, BBR was shown to regulate the proliferation and adhesion of RA-FLS cells through the RAS/MAPK/FOXO/HIF-1 signaling pathway, as well as reduce the expression of matrix metalloproteinase (MMP)-1, MMP-3, RANKL, and TNF-α, thereby combating rheumatoid arthriti (Li et al., 2023). The anti-arthritic action of BBR may be related to AMPK activation. By activating AMPK, BBR downregulated the expression of mTORC1/HIF-1α, thereby regulating the polarization of macrophages from the pro-inflammatory M1 type to the anti-inflammatory M2 type. This suppressed the glycolytic capacity of M1 macrophages, thus exerting an anti-arthritic effect (Cheng et al., 2023). Notably, in rheumatoid arthritis, macrophages and T cells jointly mediate the inflammatory cascade response. Exosomes derived from M1 macrophages can deliver miR-155 to CD4^+^ T cells to mediate immune metabolic disorder, and BBR reportedly inhibits this delivery process, thereby regulating the immune metabolism of CD4^+^ T cells (Cai et al., 2024).

BBR can also impact the metabolic mechanisms of certain oral and dental diseases. BBR was found to play a role in suppressing the growth of *Porphyromonas gingivalis* and the activity of gingival proteases. Osteoblasts and osteoclasts, as the main functional cells involved in bone formation, work in conjunction to regulate bone metabolism. BBR reportedly promotes osteogenic differentiation through the Wnt/β-catenin signaling pathway and enhances the expression of osteogenic-related genes in bone mesenchymal stem cells, thereby demonstrating its therapeutic potential to promote periodontal tissue regeneration (Zhang et al., 2019). The Wnt/β-catenin signaling pathway, as a classic route for BBR-stimulated osteogenic differentiation, promotes the differentiation of bone marrow mesenchymal stem cells into the osteogenic lineage (Tao et al., 2016) and regulates the development of dental tissues and the self-renewal of stem cells (Song et al., 2017; Wang et al., 2018). BBR also promotes odontogenic cell differentiation through the Wnt/β-catenin signaling pathway, where odontogenic cells, derived from dental pulp stem cells, are crucial in repairing dentin and protecting the deeper dental pulp in tooth lesions. This evidence supports the potential application of BBR in treating dental caries. Additionally, treatment with BBR was shown to repair bone loss in rats with apical periodontitis by restoring the balance between bone resorption and formation while also reducing the activity and quantity of osteoclasts and enhancing osteoblast differentiation. More importantly, BBR increased the expression of lysyl oxidase and decreased the levels of MMPs, which play important roles in extracellular matrix biosynthesis, thus promoting the stability of the extracellular matrix in periapical periodontitis (Cui et al., 2023), which is crucial for bone homeostasis. Collectively, these results indicate that BBR regulates bone metabolism in periodontitis and apical periodontitis, repairs bone damage by altering gingiva and extracellular matrix enzyme activity, and promotes odontogenic cell differentiation and bone homeostasis.

## 3 Key targets and clinical value of BBR in regulating metabolism

### 3.1 Molecular docking of key targets of BBR regulation of metabolism

**FIGURE 4:**
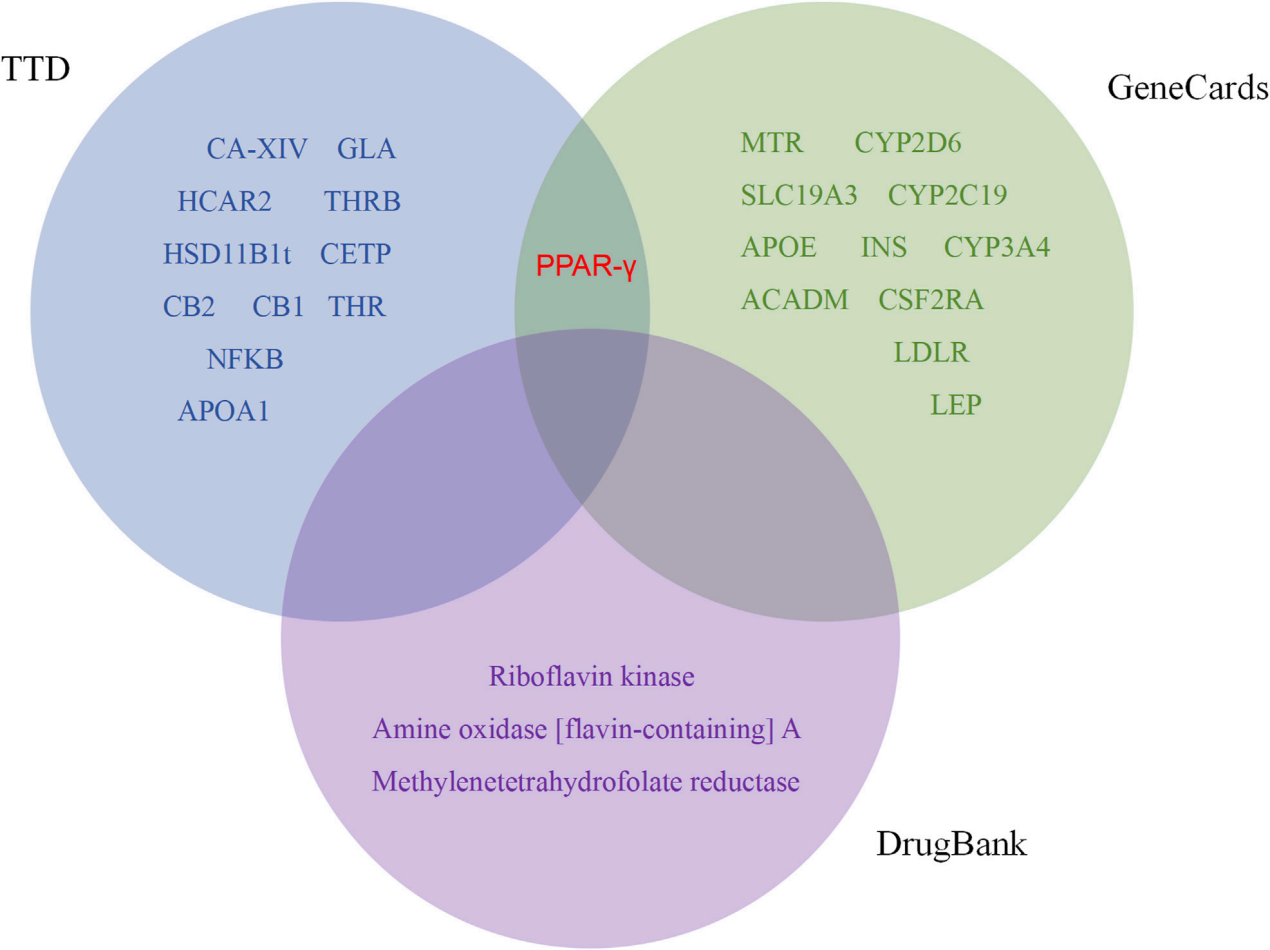
Venn diagram of berberine and metabolic related targets.

**FIGURE 5:**
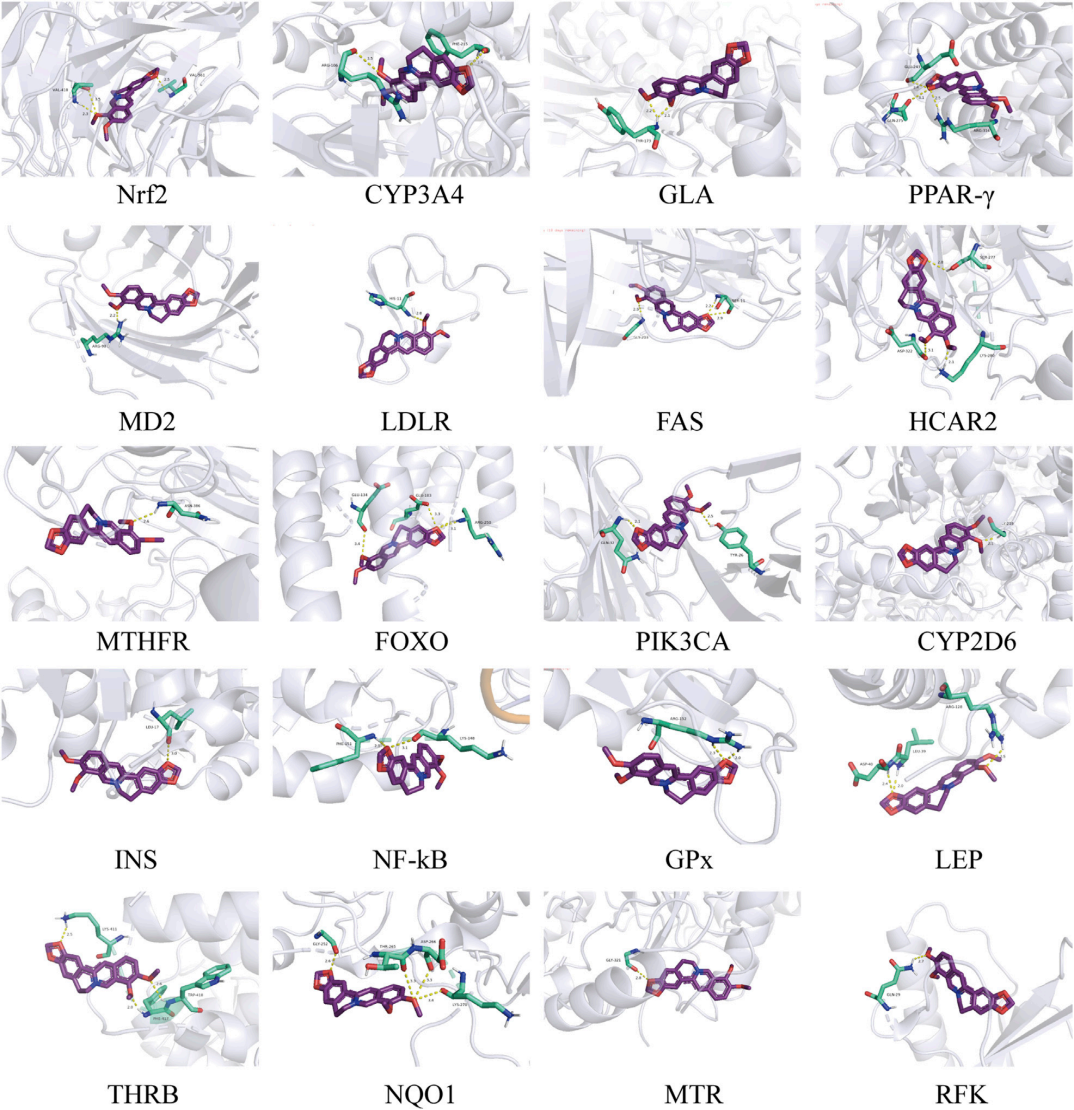
The binding results of BBR and metabolism-related targets.

In previous explanations of animal and cell studies that have been validated, it has been partially demonstrated that BBR plays a metabolic role and mechanism in systemic regulation. It has been found that BBR exerts metabolic regulatory effects by targeting certain cells or regulatory protein targets. We further searched metabolism-related targets through TTD, DrugBank, and GeneCards, and included cutting-edge literature research to summarize potential metabolic targets. In addition, the binding of BBR to these targets was evaluated by molecular docking. Among them, 26 targets capable of docking with BBR were retrieved from three databases (Figure 4). PPAR-γ was identified as a common target in the TTD and GeneCards databases. Based on docking and metabolic correlation, we selected the top 20 targets with the highest affinity scores (Table 2), and BBR interacted with these macromolecular proteins (Figure 5) (Supplementary Figure S1). Although certain important signaling molecules, such as NFKB, LDLR, and APOA1, were detected in only one database and were not identified as common targets, they were still considered to have important metabolic effects and mechanisms in the above-mentioned animal and cell studies exploring BBR intervention. We further merged and compared the top 20 scoring molecules with the targets mentioned earlier, revealing 5 targets that were predicted by molecular docking and validated by experiments, namely, Nrf2, INS, PPAR-γ, LDLR, and NF-KB. Further validation confirmed the regulatory effects of these targets in the treatment of metabolic diseases such as diabetes, obesity, and hyperlipidemia by BBR.

**TABLE 2:** Binding energy of the top 20 docking scores of BBR and the amino acid residues to the metabolism-related targets.

	Protein	PDB ID	Docking score (kcal.mol)	Binding feature
1	Nrf2	7.52	5WFV	VAL-418, VAL-561
2	CYP3A4	7.21	1TQN	ARG-106, PHE-215
3	GLA	6.72	3GXP	TYR-173
4	PPAR-γ	6.64	1FM9	GLU-243, GLN-275, ARG-316
5	MD2	6.62	2E56	ARG-90
6	LDLR	6.5	1AJJ	HIS-11
7	FAS	6.45	3THM	GLY-203, SER-11
8	HCAR2	6.45	7XK2	ASP-322, SER-277, LYS-280
9	MTHFR	6.43	6FCX	ASN-386
10	FOXO3	6.36	7v9b	GLU-134, GLU-183, ARG-250
11	PIK3CA	6.27	7L1D	GLN-32, TYR-26
12	CYP2D6	6.23	3TBG	GLY-218
13	INS	6.2	1QIY	LEU-17
14	NF-KB	6.2	1SVC	PHE-151, LYS-148
15	GPx4	6.19	2OBI	ARG-152
16	LEP	6.08	1AX8	ASP-40, LEU-39, ARG-128
17	THRB	6.06	1N46	LYS-411, PHE-417, TRP-418
18	NQO1	6.05	2F1O	GLY-252, THR-265, ASP-266, LYS-270
19	MTR	6.03	4CCZ	GLY-321
20	RFK	6	1NB0	GLN-29

### 3.2 Clinical value of BBR

At present, the therapeutic effect of BBR on metabolic disorders in clinical practice has been well described. The relatively clear effects include reducing blood glucose level, alleviating lipid disorders, improving insulin resistance, alleviating inflammation and oxidative stress. It has been shown in the previous discussion that BBR stimulates insulin secretion, reduces glucose transport, and inhibits hepatic gluconeogenesis by acting on GLP1, GLUT2, MPC1, TAS2R38, SIRT1, and other targets, which partially reveals the possible mechanism of BBR in treating diabetes. There are clinical trial reports that BBR can significantly improve serum fasting blood glucose and HbA1c levels in T2DM patients, and help restore pancreatic island function. In addition, BBR also inhibits the formation of foam cells, regulates lipid metabolism, and inhibits atherosclerosis related inflammatory reactions by regulating the levels of visfatin, oxLDL, PON1, APOA1, GPD2, ABCG1, and AMPK, which may have good clinical value for the treatment of cardiovascular diseases. A randomized, placebo-controlled, multicenter clinical trial showed that BBR could improve postprandial total cholesterol and low-density lipoprotein cholesterol levels in type 2 diabetes patients, thereby reducing the risk of cardiovascular disease in dyslipidemic patients with type 2 diabetes (Wang et al., 2022). This further confirmed from a clinical perspective that BBR can improve metabolic abnormalities such as blood glucose, blood lipid, and insulin resistance in T2DM patients, and its therapeutic applications cover type 2 diabetes and cardiovascular diseases. BBR also regulates lipid metabolism and steatosis of hepatocytes by regulating PCSK9, LDL-R, SIRT1, and SREBP2 levels, which suggests that BBR may have potential in the clinical application of non-alcoholic fatty liver disease and hyperlipidemia. A randomized controlled trial showed that BBR can reduce liver fat content, improve blood lipid levels, and reduce body weight in nonalcoholic fatty liver disease (NAFLD) patients (Yan et al., 2015). In conclusion, BBR has shown a good clinical potential to alleviate diabetes, cardiovascular disease, NAFLD, and obesity in clinical trials, which is related to reducing blood glucose, alleviating lipid disorders, improving insulin resistance, and alleviating inflammation. However, its exact mechanism needs to be further confirmed by experiments.

**FIGURE 6:**
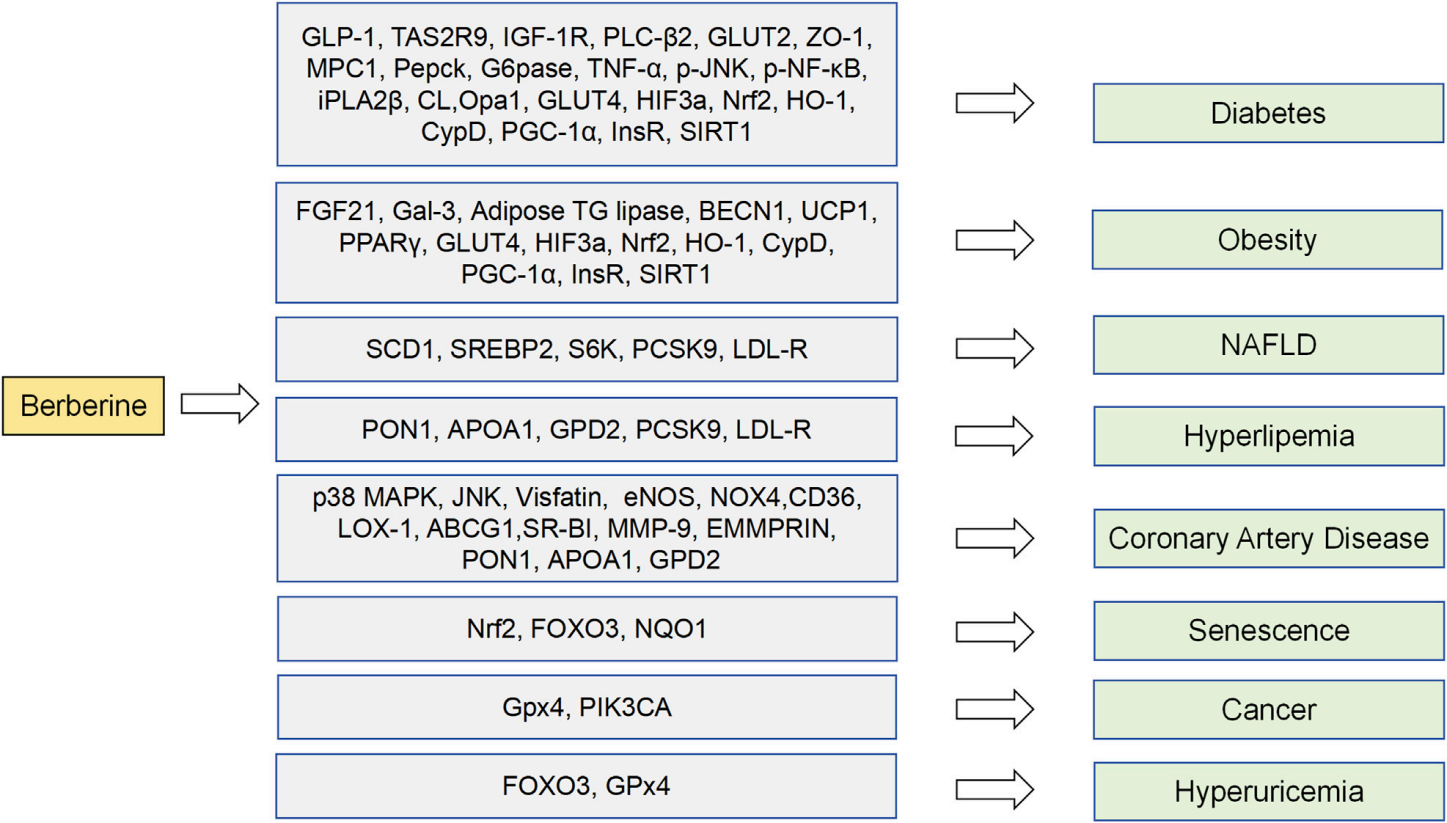
Metabolism related protein targets and clinical disease potential.

Based on metabolic related disease target database and molecular docking results mentioned above, the clinical therapeutic potential of BBR on potential targets was further predicted. We found that FOXO3, Nrf2, NQO1, Gpx4 and PIK3CA are not only related to regulating metabolic diseases, but also may have therapeutic effects on aging, cancer, or hyperuricemia (Figure 6). As a mediator of antioxidant responses and DNA damage repair, FOXO 3 is thought to have the potential to combat aging and stimulate tissue regeneration (de Keizer, 2017). BBR may play a role in preventing and treating aging by regulating FOXO3. Activation of AMPK can increase FOXO3 transcriptional activity. Notably, BBR can activate AMPK (Xu et al., 2017). Based on this, we suggest that BBR has a great possibility of inhibiting aging by regulating FOXO3 activity. Similarly, Nrf2 and NQO1 are also associated with antioxidant and aging control. Nrf2 promotes NQO1 transcription and expression by combining to NQO1 gene promoter regions. As an important oxidoreductase, NQO1 can reduce quinone to hydroquinone and reduce harmful oxidation of quinone (Chen et al., 2000). NQO1 is positively correlated with life span extension in animal models and may contribute to caloric restriction and control aging. Whether the anti-aging effect of BBR is related to NQO1 regulation, deserves further research. New research suggests a link between cancer and metabolic disorders (Glover and Glick, 1987). In cancer therapy, promoting ferroptosis of drug-resistant cancer cells by inhibiting GPX4 is seen as a potential therapeutic strategy. It was found that BBR has a high affinity to Gpx4 through molecular docking. As a key regulator of ferroptosis, Gpx4 may be a potential target for BBR to promote ferroptosis and exert anti-cancer effects. PIK3CA gene mutation and its resulting protein dysfunction can trigger dysregulation of PI3K/Akt signaling pathway, thereby promoting tumor formation and development. BBR has been shown to regulate the PI3K/Akt signaling pathway to promote apoptosis in thyroid cancer cells. Therefore, whether BBR can affect the activation of PI3K and Akt in cancer cells with PIK3CA mutations and inhibit PI3K phosphorylation is worthy of further research. In a mouse model of hyperuricemia after drug intervention, it was found that reducing uric acid was accompanied by upregulation of FOXO3 protein expression and changes of glutathione peroxidase levels. It is suggested that the regulation of FOXO3 and GPx4 may be related to the therapeutic mechanism of improving hyperuricemia, but there is no clinical evidence to prove it (An et al., 2023).

## 4 Conclusion and future perspective

In summary, BBR has a positive therapeutic effect and broad application prospects in the pathological changes of human-related metabolic diseases and chronic diseases by participating in the metabolic activities of various systems. Many signaling pathways mediated by target receptors play a crucial role in regulating system metabolism and target cell fate. Targeting these pathways in metabolic regulation may bring new strategies for the treatment of metabolic diseases. Our molecular docking study further predicts that BBR may act on specific potential targets, such as FOXO3, Nrf2, NQO1, Gpx4, PIK3CA, and GPx, which are promising molecular targets for treating aging, cancer, or hyperuricemia. However, the limitations of this study need to be addressed. As BBR itself is a pan-assay interfering metabolite, it may possess the capacity to bind to multiple proteins. There is a lack of correlation between the binding states predicted by molecular docking and the actual biological effects generated by molecules. The metabolic mechanism of BBR involves several broad-spectrum signaling pathways, several of which are yet to be comprehensively elucidated, and research on related signal transduction pathways remains poorly clarified, necessitating further animal and cell experiments. Currently, there is a lack of research on the therapeutic mechanisms of BBR at the molecular and cellular levels in humans, and it is necessary to fully utilize metabolomics, transcriptomics, or proteomics technologies for further elucidation. Furthermore, the appropriate BBR dose remains unclear, and the effects of different dosages on systemic metabolism remain poorly explored. In the future, additional high-quality basic and clinical trials are needed to establish the dose-response effects of BBR, which will also facilitate the development of BBR and related products. In future research endeavors, key priorities include in-depth investigations into BBR-mediated molecular mechanisms that regulate metabolism through potential targets, the use of novel research techniques to identify scientific evidence of ligand-receptor interactions of potential targets, and the development of new biomarkers to explore the possibility and specificity of BBR in treating chronic diseases, as well as for specific targeting strategies.

## Figures and Tables

**Table:** 

**ABCG1**	ATP-binding cassette transporter G1
**AKT1**	serine/threonine-protein kinase 1
**AMPK**	AMP-activated protein kinase
**APOA1**	apolipoprotein A1
**BECN1**	Beclin 1
**cAMP**	cyclic adenosine 3′-5′ monophosphate
**CL**	cardiolipin
**CypD**	cyclophilin D
**eNOS**	endothelial nitric oxide synthase
**ER**	endoplasmic reticulum
**FGF**	fibroblast growth factor
**FoxO**	forkhead box O
**Gal-3**	galectin-3
**GLP-1**	glucagon-like peptide-1
**GLUT**	glucose transporter
**GPD2**	glycerol-3-phosphate dehydrogenase 2
**Gpx**	Glutathione Peroxidase
**HFD**	high-fat diet
**HNF1α**	hepatic nuclear factor 1 alpha
**iPLA2β**	calcium-independent phospholipase A2β
**InsR**	Insulin Receptor
**JNK**	c-Jun N-terminal kinase
**KCNH6**	potassium voltage-gated channel subfamily H member 6
**LDL-C**	low-density lipoprotein cholesterol
**LDL-R**	low-density lipoprotein receptor
**LOX**	lectin-like oxLDL receptor
**MAPK**	mitogen-activated protein kinase
**MCP-1**	monocyte chemoattractant protein-1
**miR**	micro-RNA
**MPC**	mitochondrial pyruvate carrier
**mRNA**	the messenger ribonucleic acid
**mTOR**	mammalian target of rapamycin
**NAFLD**	nonalcoholic fatty liver disease
**NOX**	nicotinamide adenine dinucleotide phosphate oxidase
**NQO1**	NAD (P) H quinone oxidoreductase 1
**NF-κB**	nuclear factor kappa-light-chain-enhancer of activated B cells
**Nrf2**	nuclear factor erythroid 2-related factor 2
**Opa1**	optic atrophy 1
**PON1**	paraoxonase-1
**PPAR**	proliferator-activated receptor
**ROS**	reactive oxygen species
**PCSK9**	proprotein convertase subtilisin/kexin type 9
**PGC-1α**	peroxisome proliferator-activated receptor-gamma coactivator 1-alpha
**PIK3CA**	phosphatidylinositol-4,5-bisphosphate 3-kinase catalytic subunit alpha
**SCD1**	stearoyl-coenzyme A desaturase 1
**SCFA**	short-chain fatty acid
**SIRT1**	Sirtuin 1
**SR**	scavenger receptors
**SREBP**	sterol regulatory element-binding protein
**TG**	triglyceride
**TLR**	Toll-like receptor
**TTD**	Therapeutic Target Database
**TNF**	tumor necrosis factor
**UCP1**	uncoupling protein-1
**WAT**	white adipose tissue.
