# Supplementary figures and images for: Molecular mechanisms, targets and clinical potential of berberine in regulating metabolism: a review focussing on databases and molecular docking studies
Source: Front Pharmacol. 2024 Jun 18;15:1368950. doi: 10.3389/fphar.2024.1368950 (PMC11217548; doi:10.3389/fphar.2024.1368950)

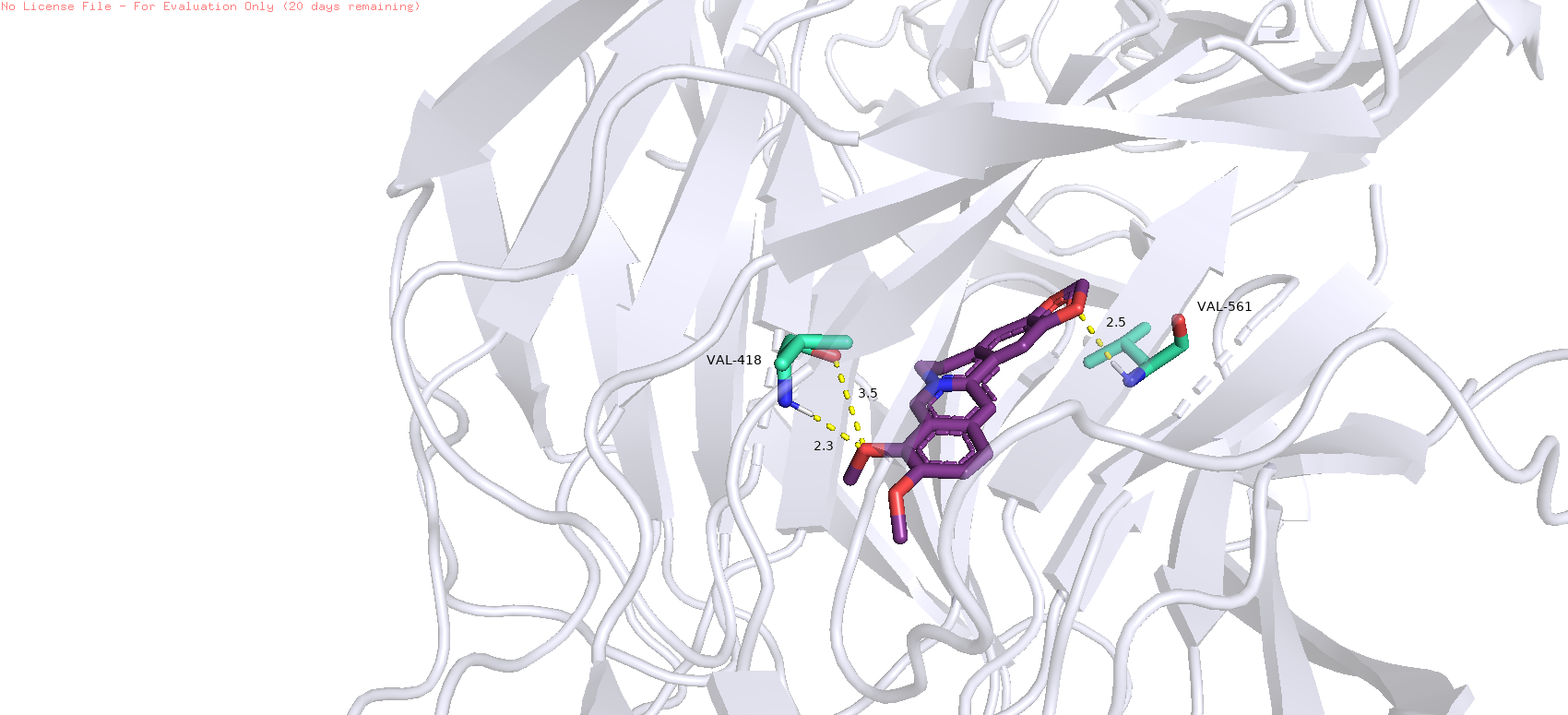

Supplement: Supplementary file 1 [file DataSheet1.ZIP › Supplementary Materials Figure S1. The binding results of BBR and metabolism-related targets/1-5WFV-ray2000.png]

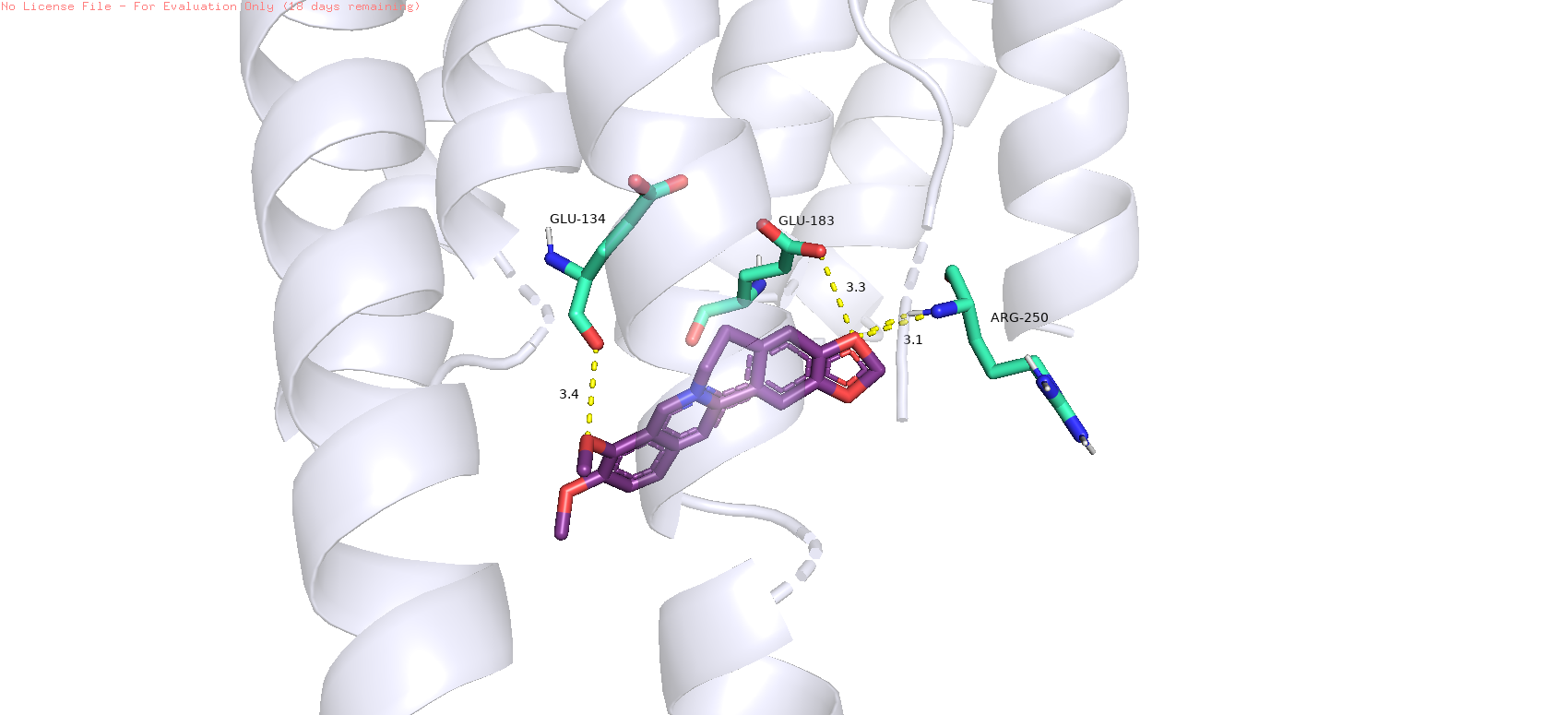

Supplement: Supplementary file 1 [file DataSheet1.ZIP › Supplementary Materials Figure S1. The binding results of BBR and metabolism-related targets/10-7V9B-ray2000.png]

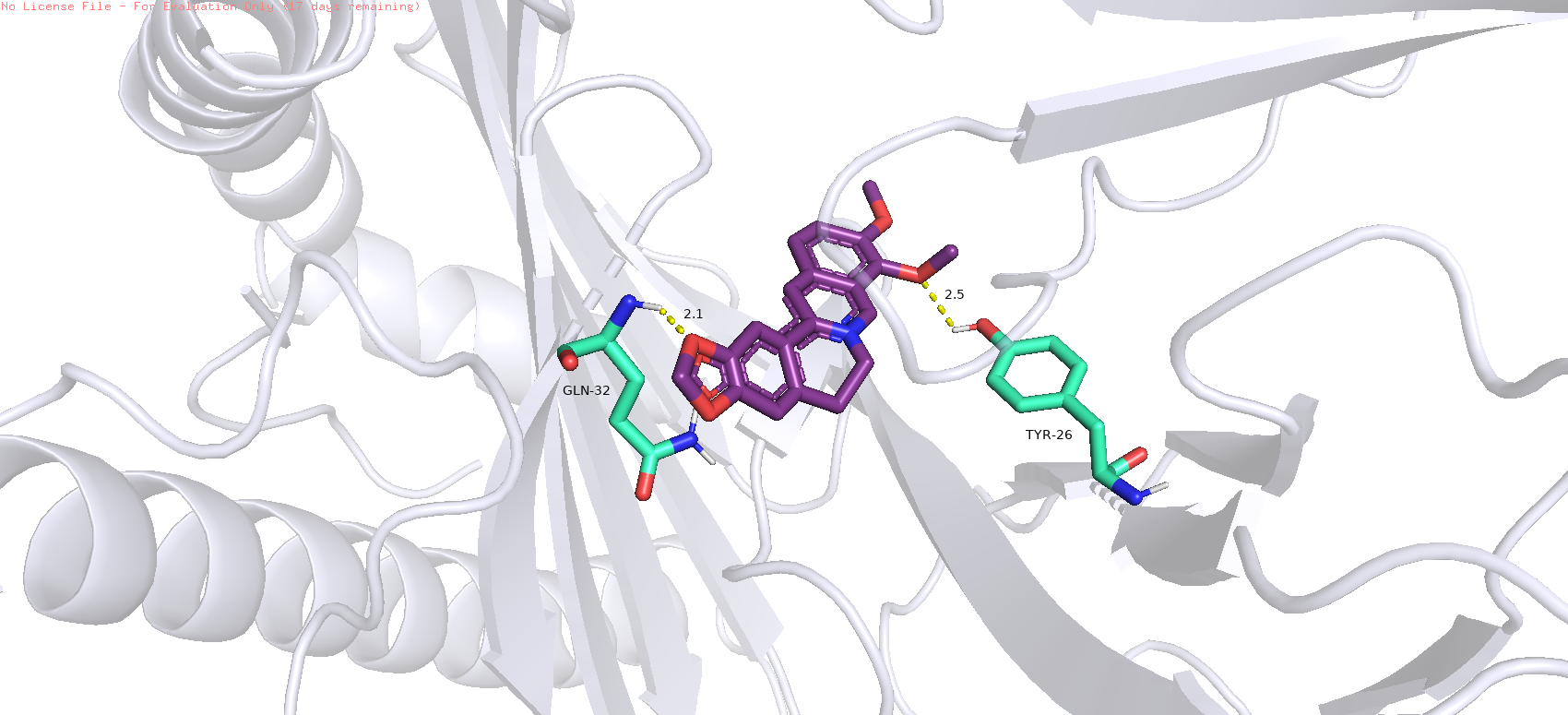

Supplement: Supplementary file 1 [file DataSheet1.ZIP › Supplementary Materials Figure S1. The binding results of BBR and metabolism-related targets/11-7L1D-ray2000.png]

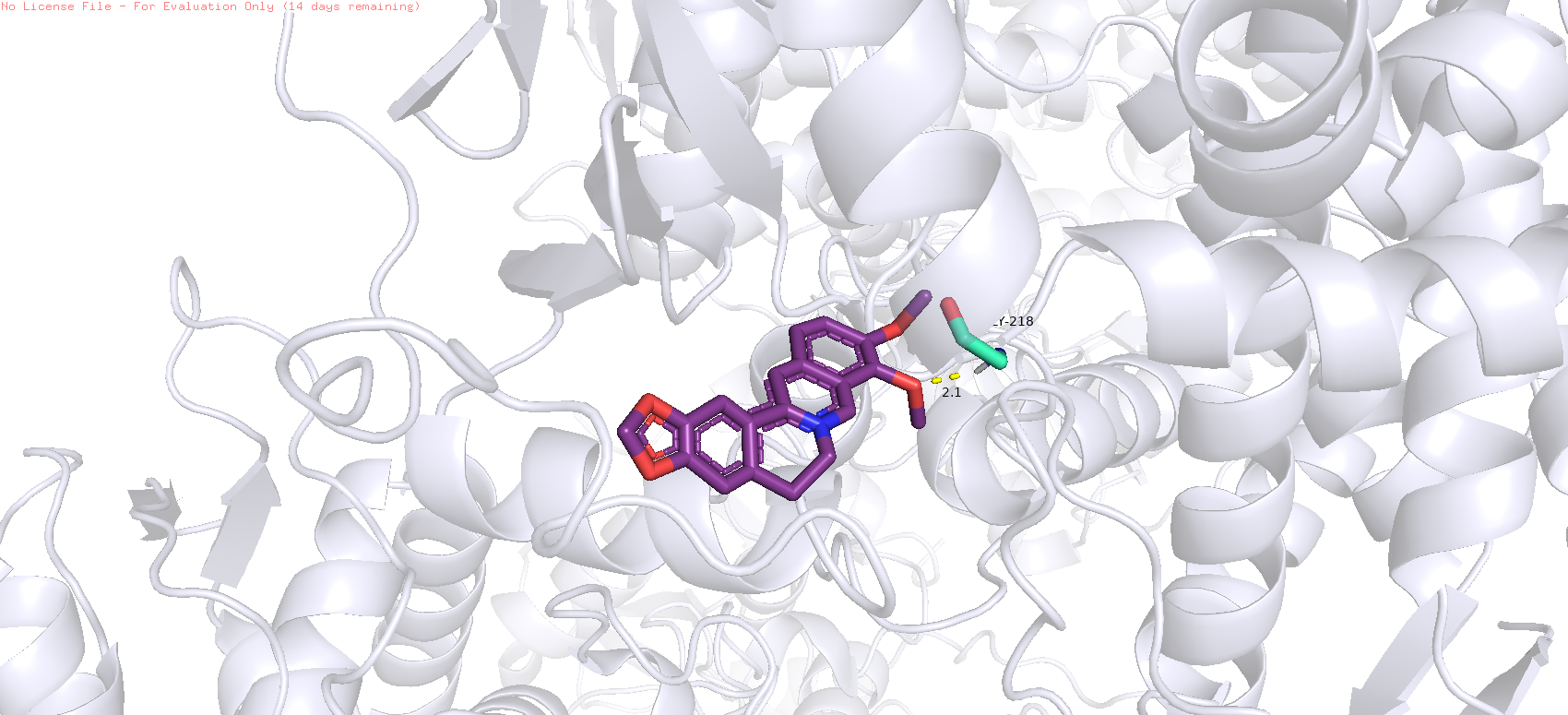

Supplement: Supplementary file 1 [file DataSheet1.ZIP › Supplementary Materials Figure S1. The binding results of BBR and metabolism-related targets/12-3TBG-xin-ray2000.png]

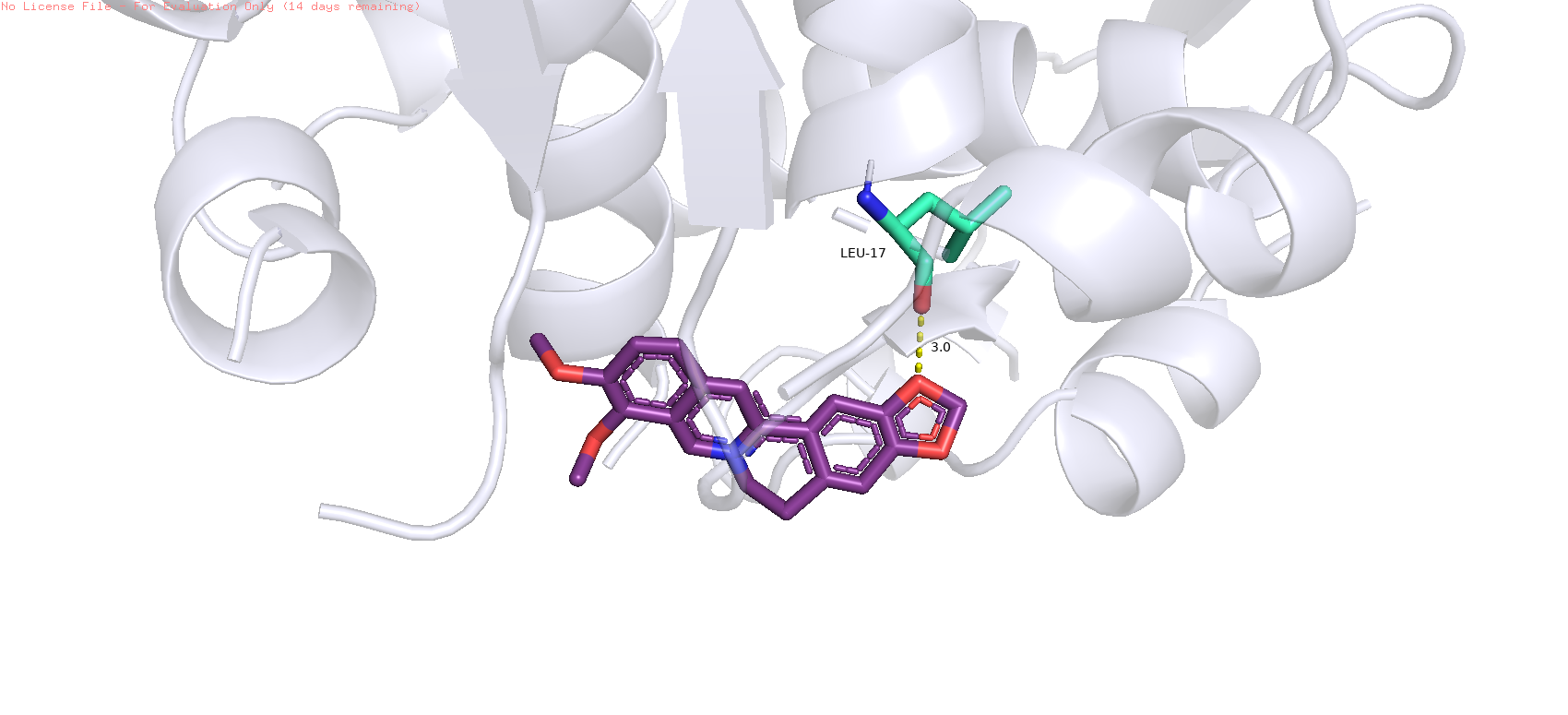

Supplement: Supplementary file 1 [file DataSheet1.ZIP › Supplementary Materials Figure S1. The binding results of BBR and metabolism-related targets/13-1QIY-xin-ray2000.png]

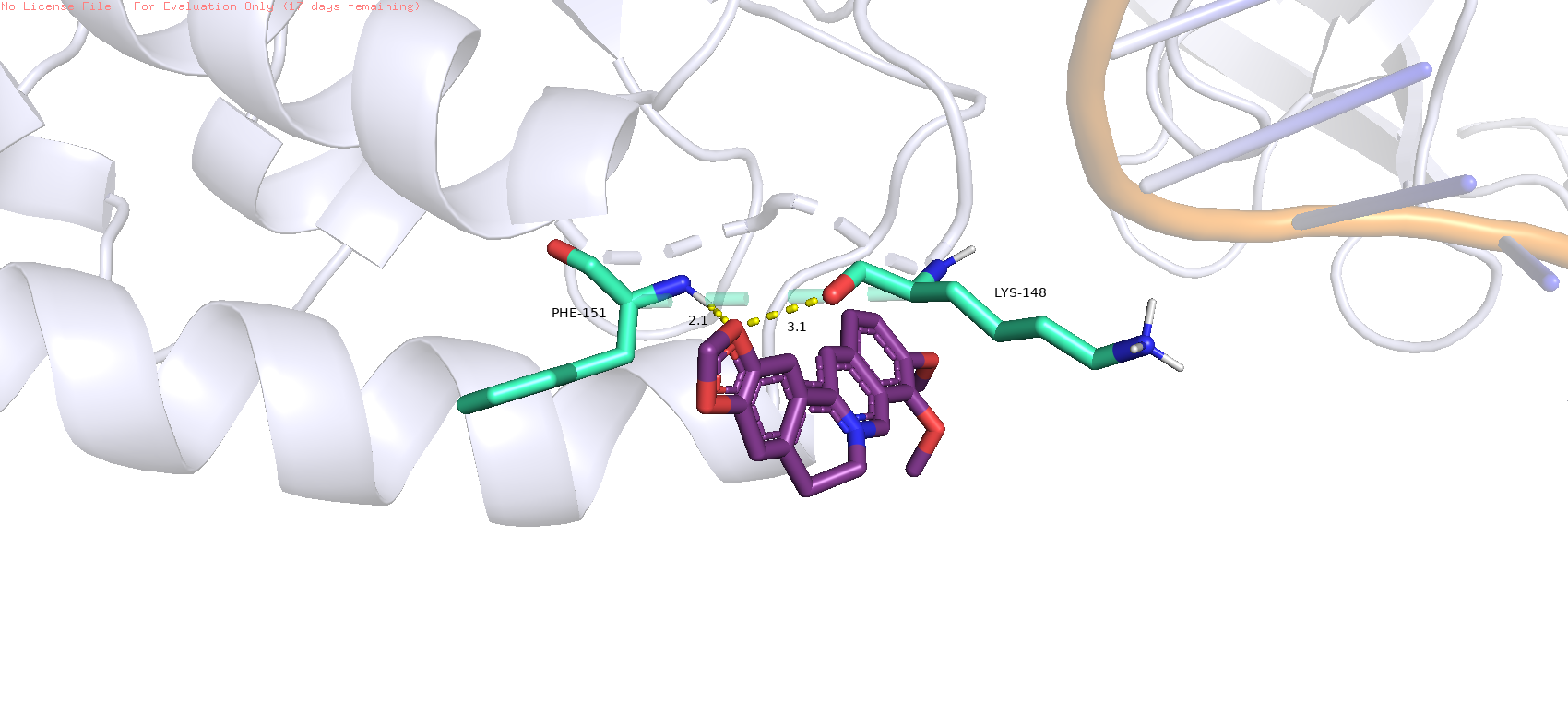

Supplement: Supplementary file 1 [file DataSheet1.ZIP › Supplementary Materials Figure S1. The binding results of BBR and metabolism-related targets/14-1SVC-ray2000.png]

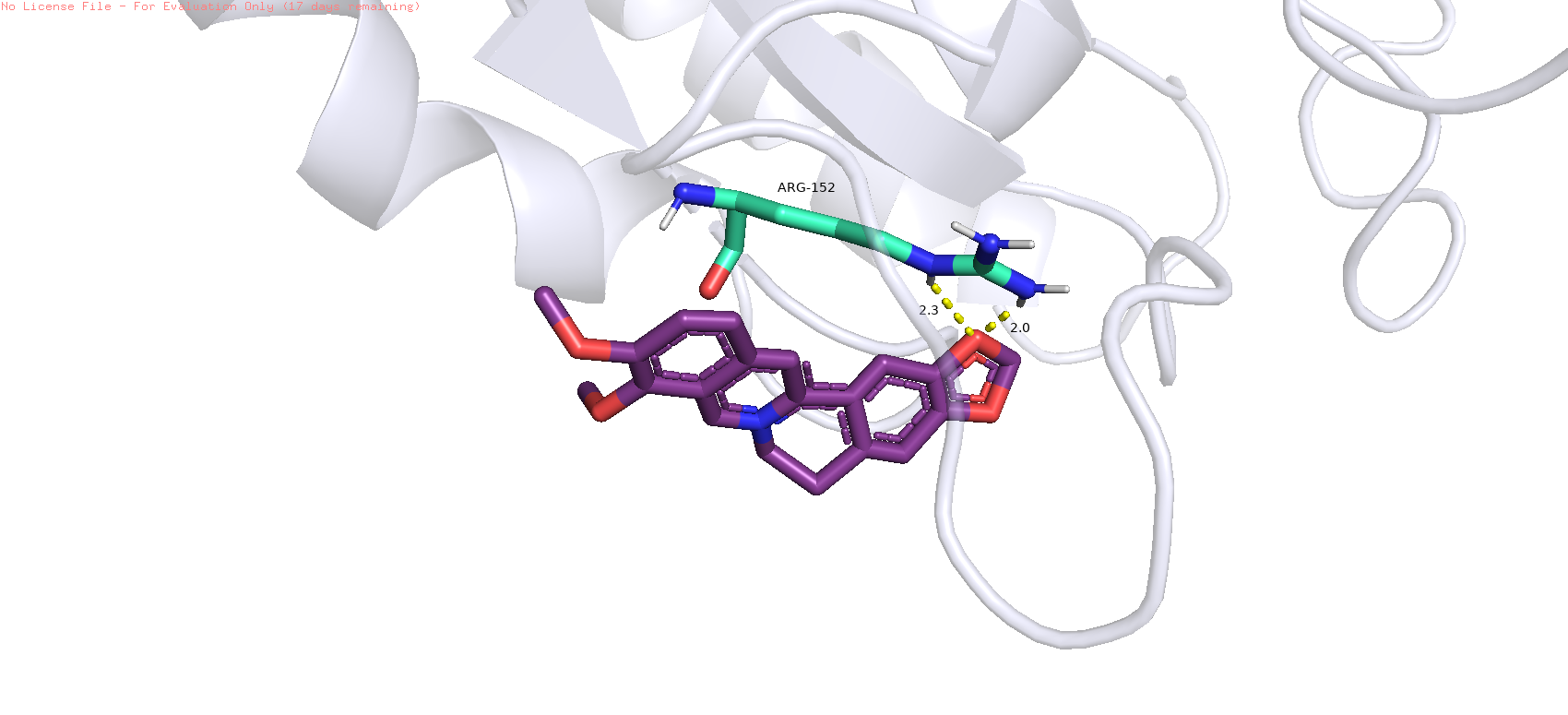

Supplement: Supplementary file 1 [file DataSheet1.ZIP › Supplementary Materials Figure S1. The binding results of BBR and metabolism-related targets/15-2OBI-ray2000.png]

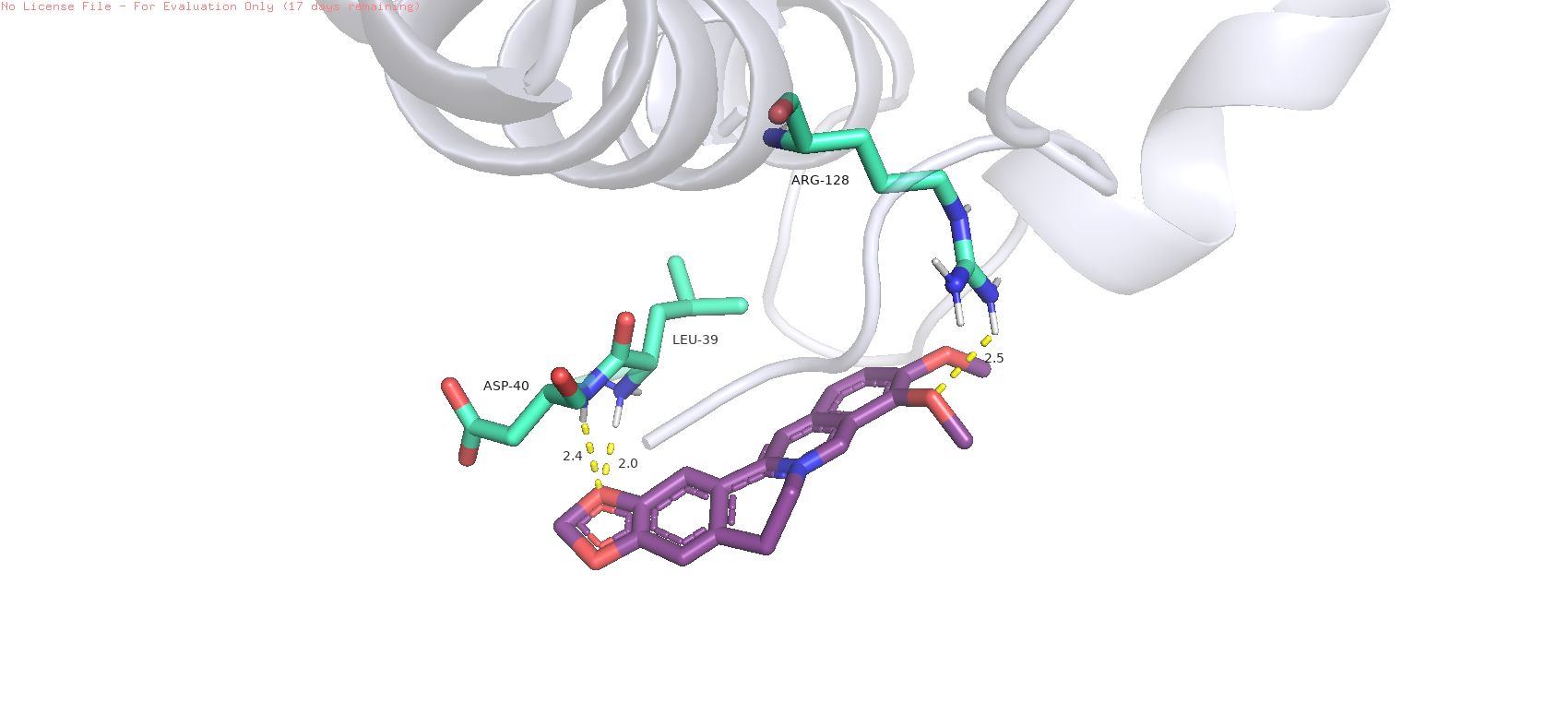

Supplement: Supplementary file 1 [file DataSheet1.ZIP › Supplementary Materials Figure S1. The binding results of BBR and metabolism-related targets/16-1AX8-ray2000.png]

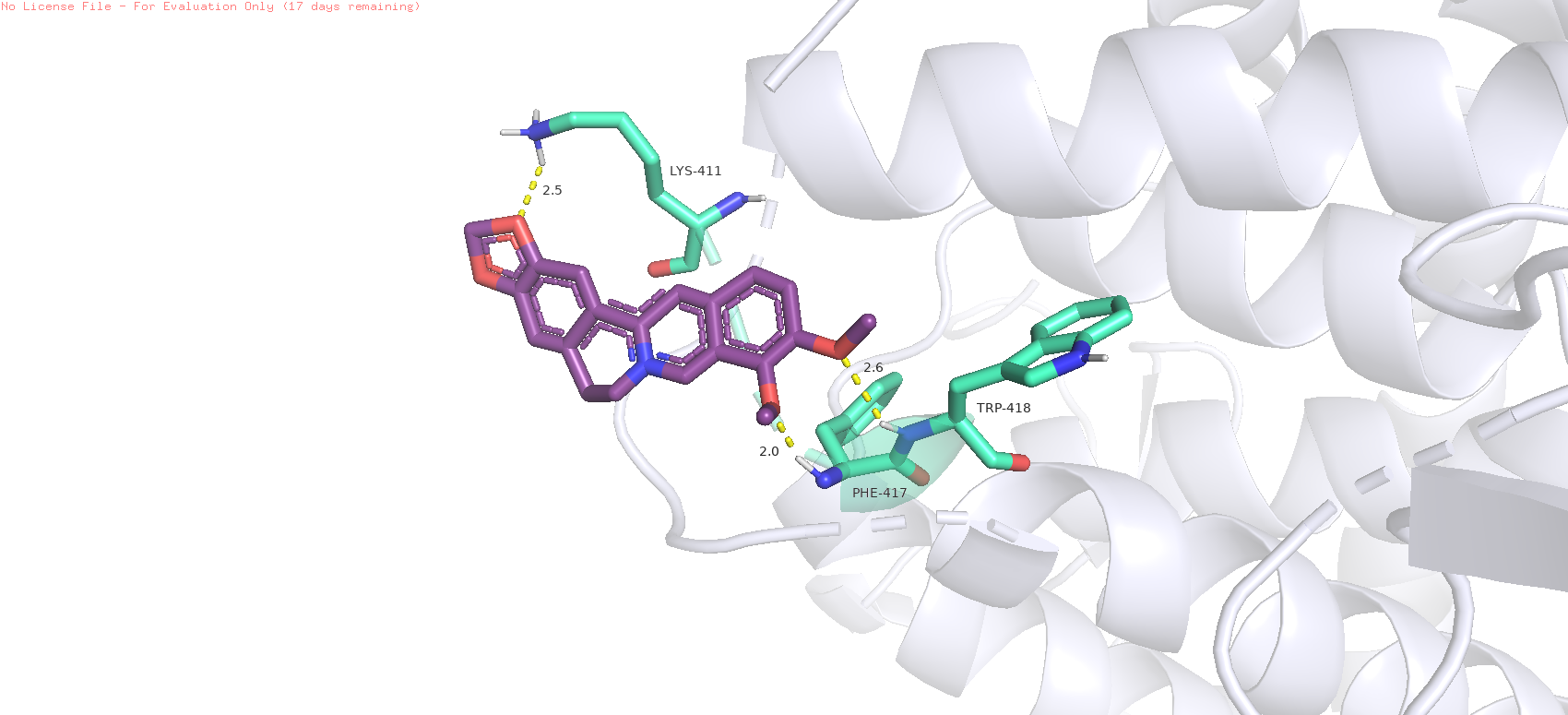

Supplement: Supplementary file 1 [file DataSheet1.ZIP › Supplementary Materials Figure S1. The binding results of BBR and metabolism-related targets/17-1N46-ray2000.png]

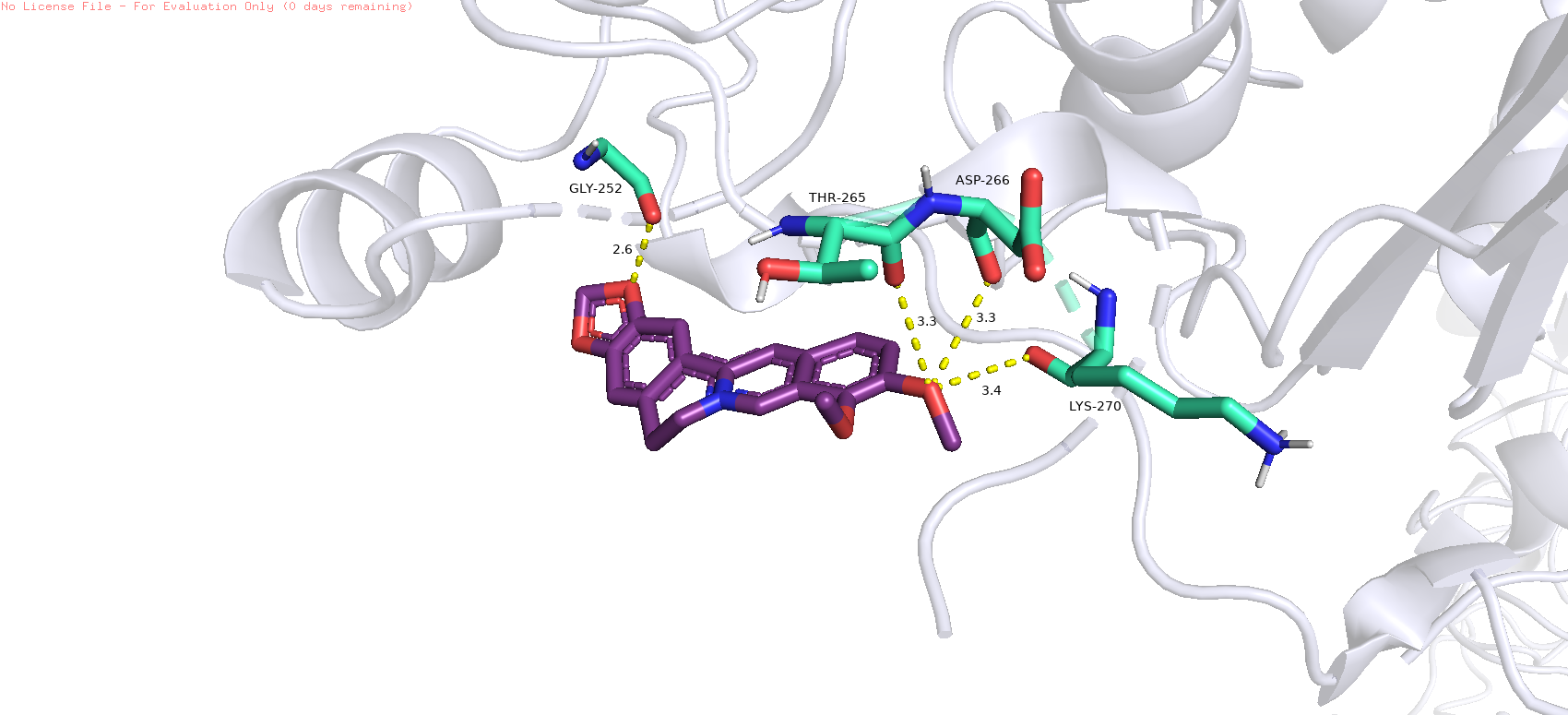

Supplement: Supplementary file 1 [file DataSheet1.ZIP › Supplementary Materials Figure S1. The binding results of BBR and metabolism-related targets/18-2F1O-ray2000.png]

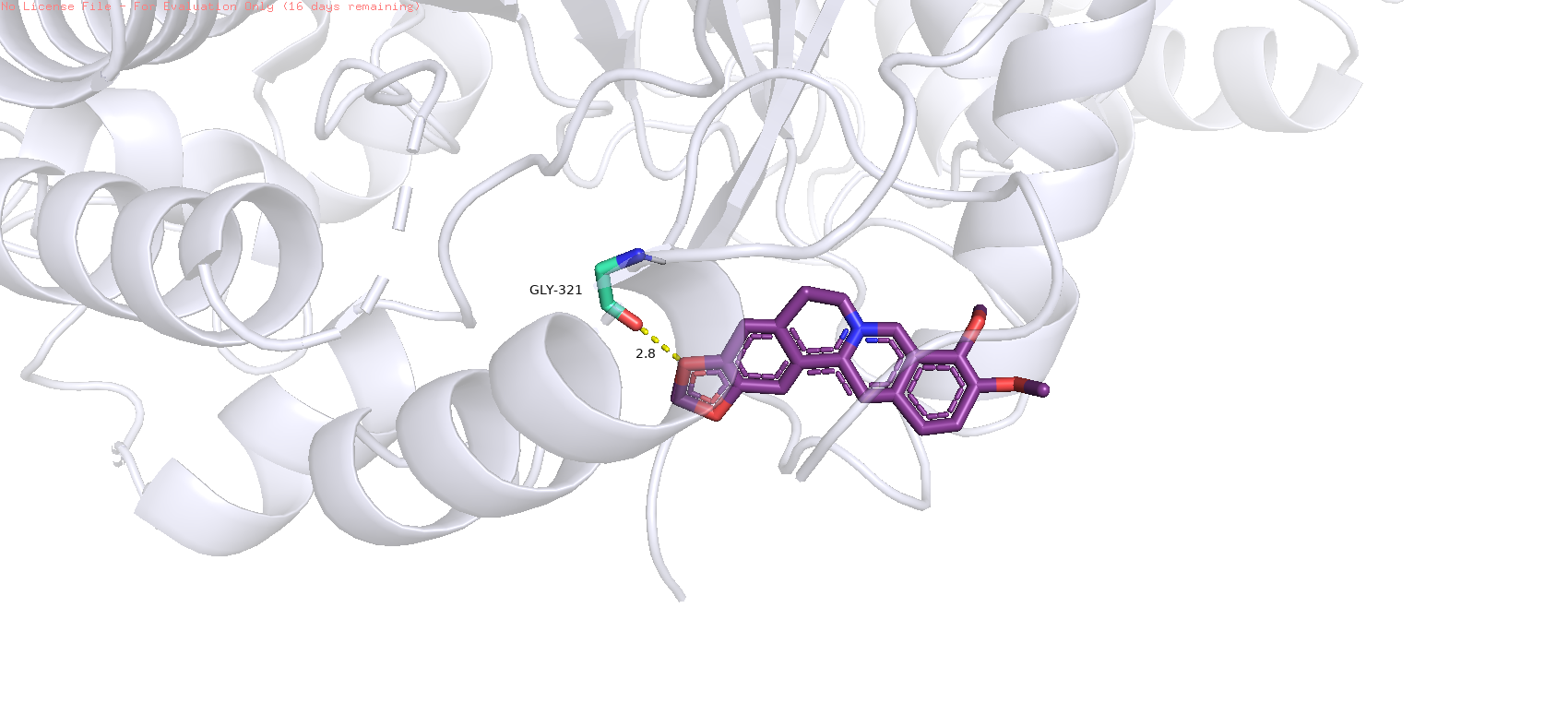

Supplement: Supplementary file 1 [file DataSheet1.ZIP › Supplementary Materials Figure S1. The binding results of BBR and metabolism-related targets/19-4CCZ-ray2000.png]

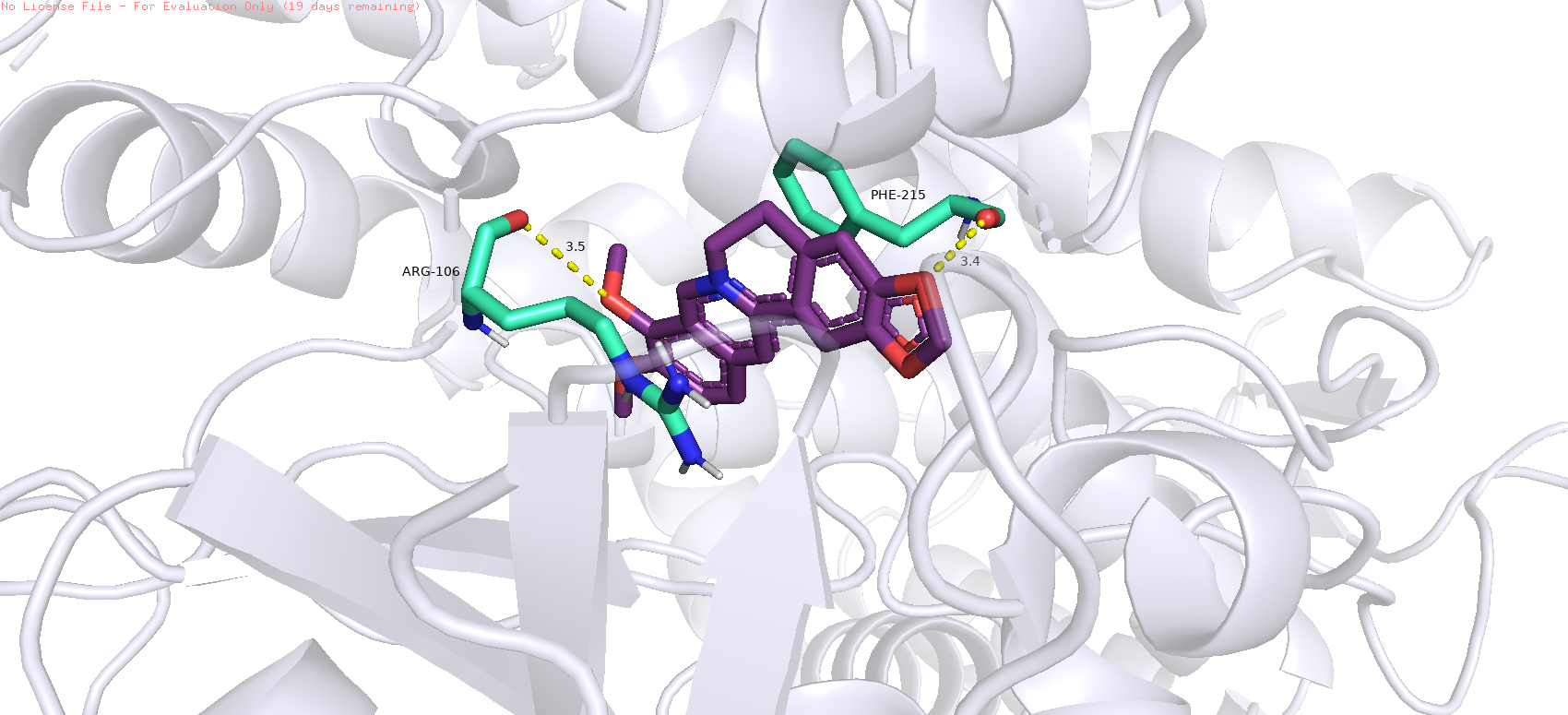

Supplement: Supplementary file 1 [file DataSheet1.ZIP › Supplementary Materials Figure S1. The binding results of BBR and metabolism-related targets/2-1TQN-ray2000.png]

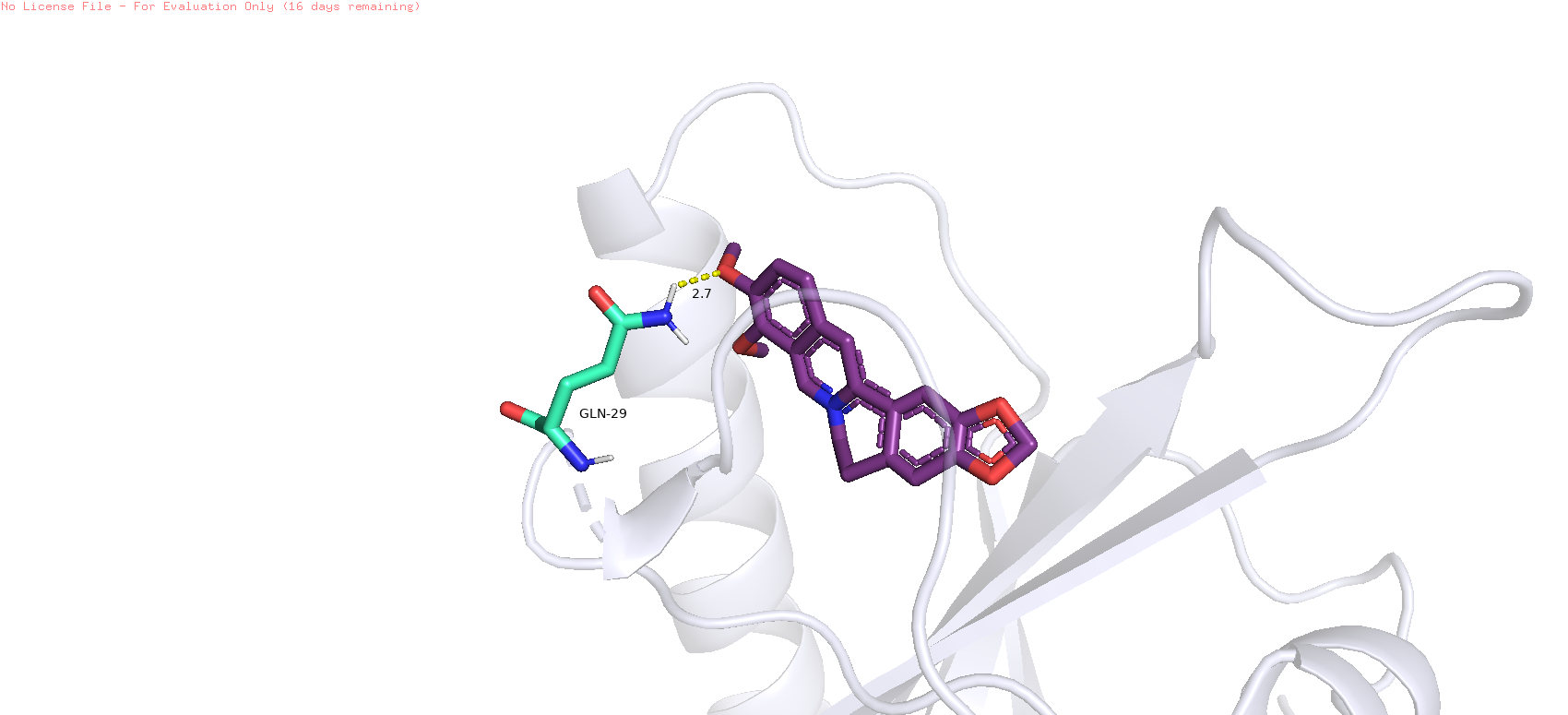

Supplement: Supplementary file 1 [file DataSheet1.ZIP › Supplementary Materials Figure S1. The binding results of BBR and metabolism-related targets/20-1NB0-ray2000.png]

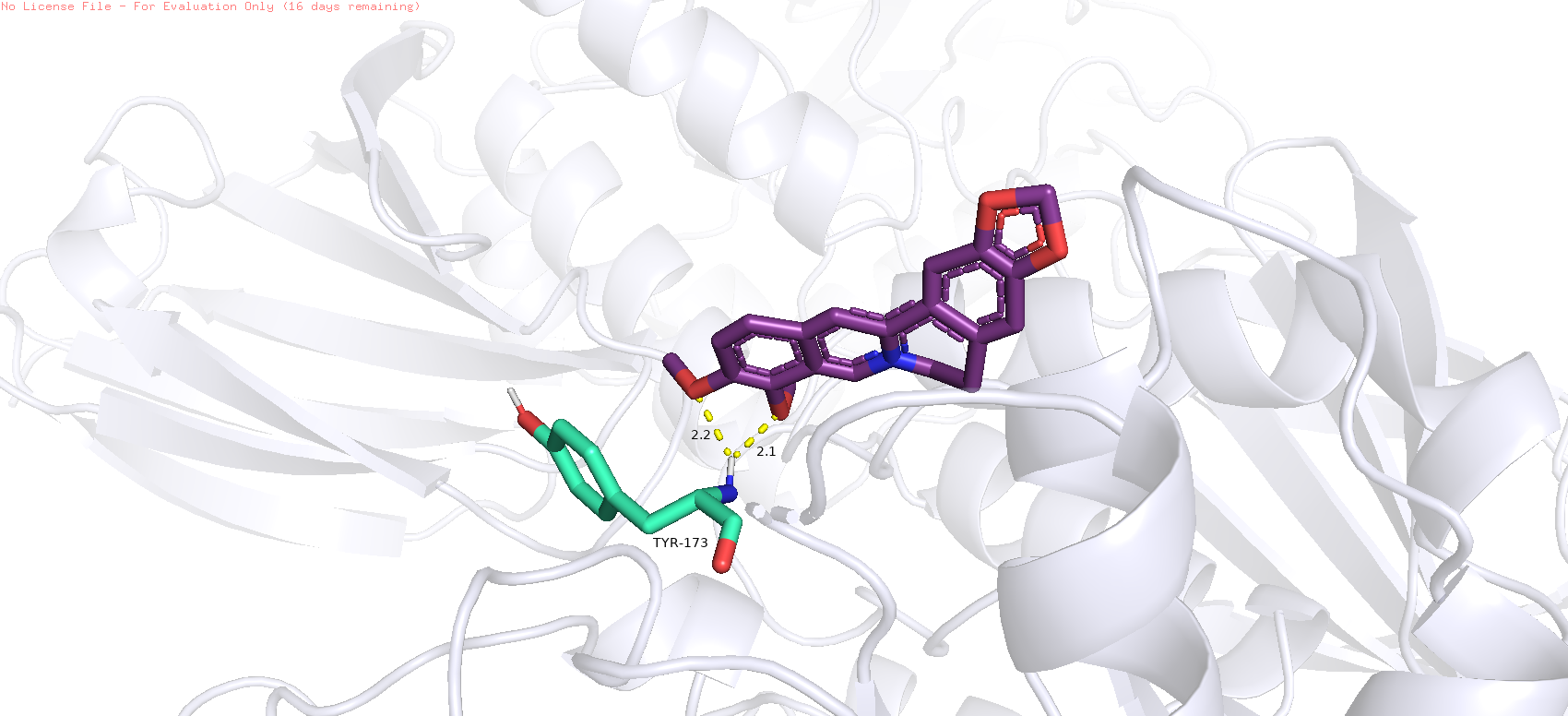

Supplement: Supplementary file 1 [file DataSheet1.ZIP › Supplementary Materials Figure S1. The binding results of BBR and metabolism-related targets/3-3GXP-ray2000.png]

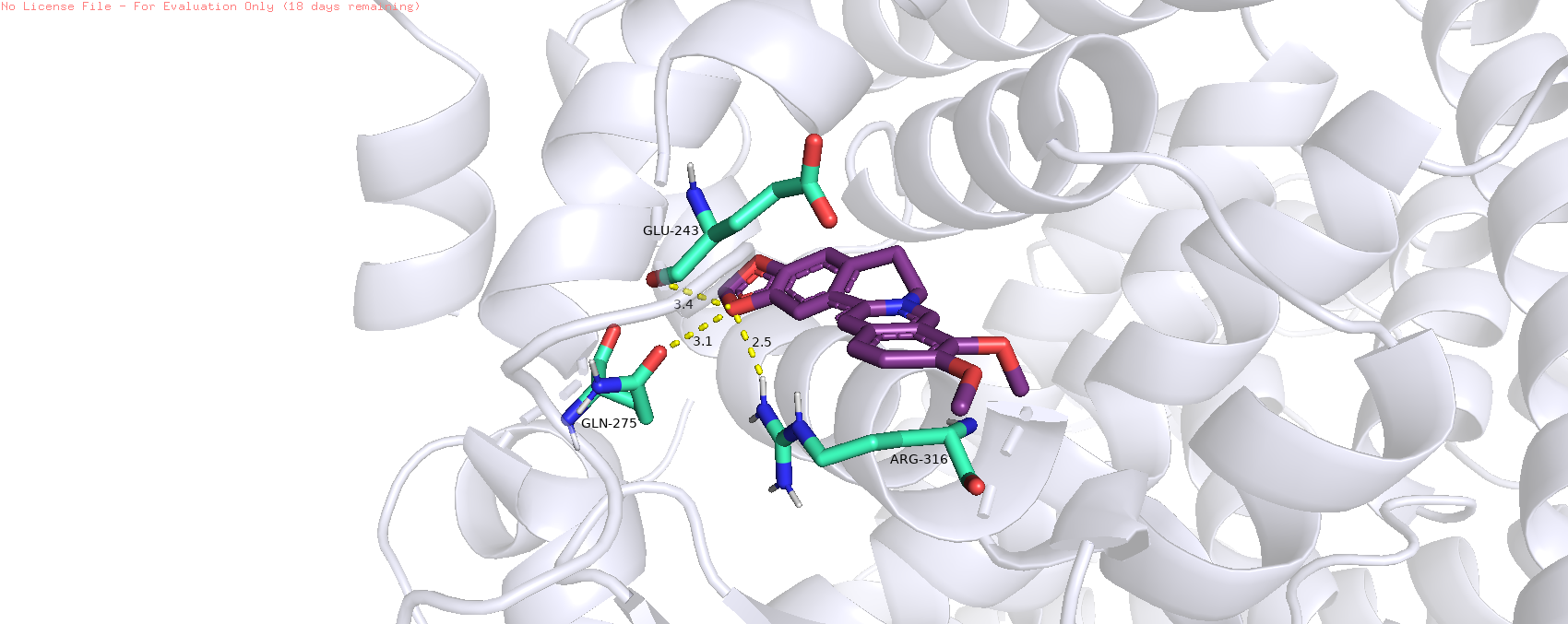

Supplement: Supplementary file 1 [file DataSheet1.ZIP › Supplementary Materials Figure S1. The binding results of BBR and metabolism-related targets/4-1FM9-ray2000.png]

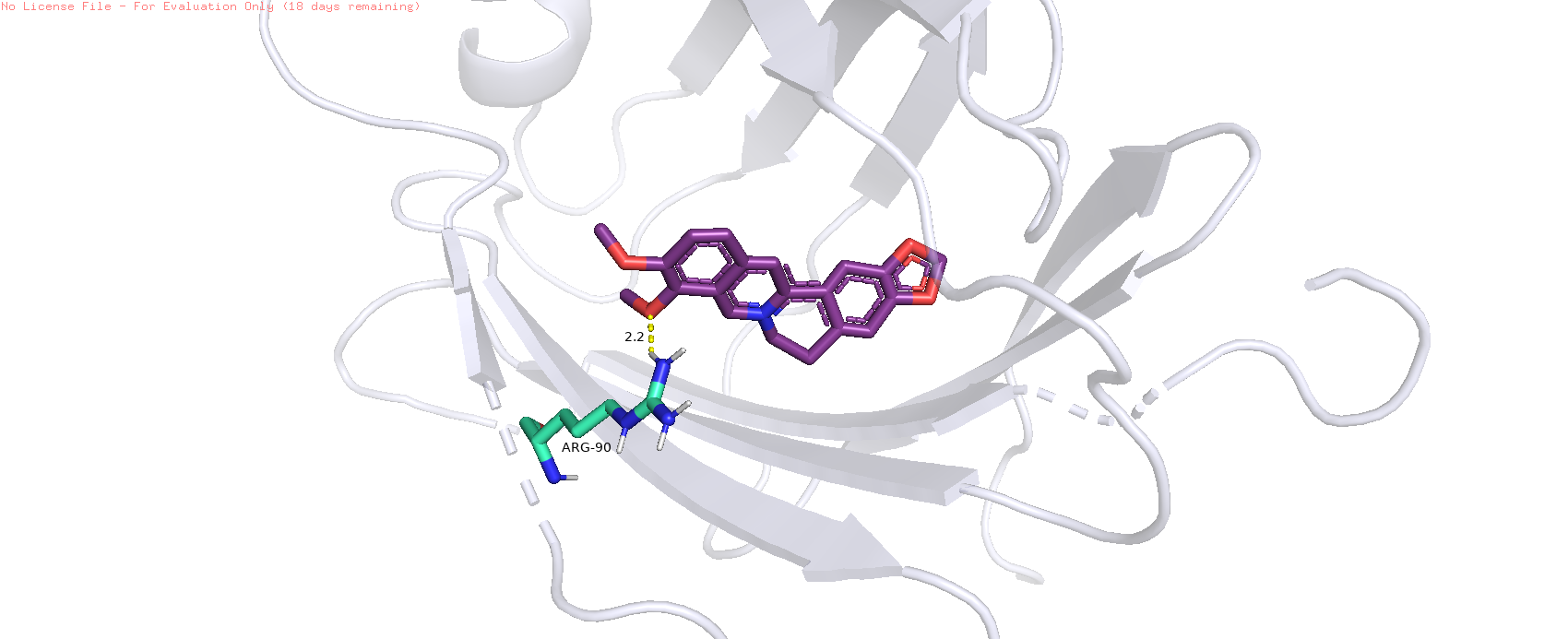

Supplement: Supplementary file 1 [file DataSheet1.ZIP › Supplementary Materials Figure S1. The binding results of BBR and metabolism-related targets/5-2E56.ray2000.png]

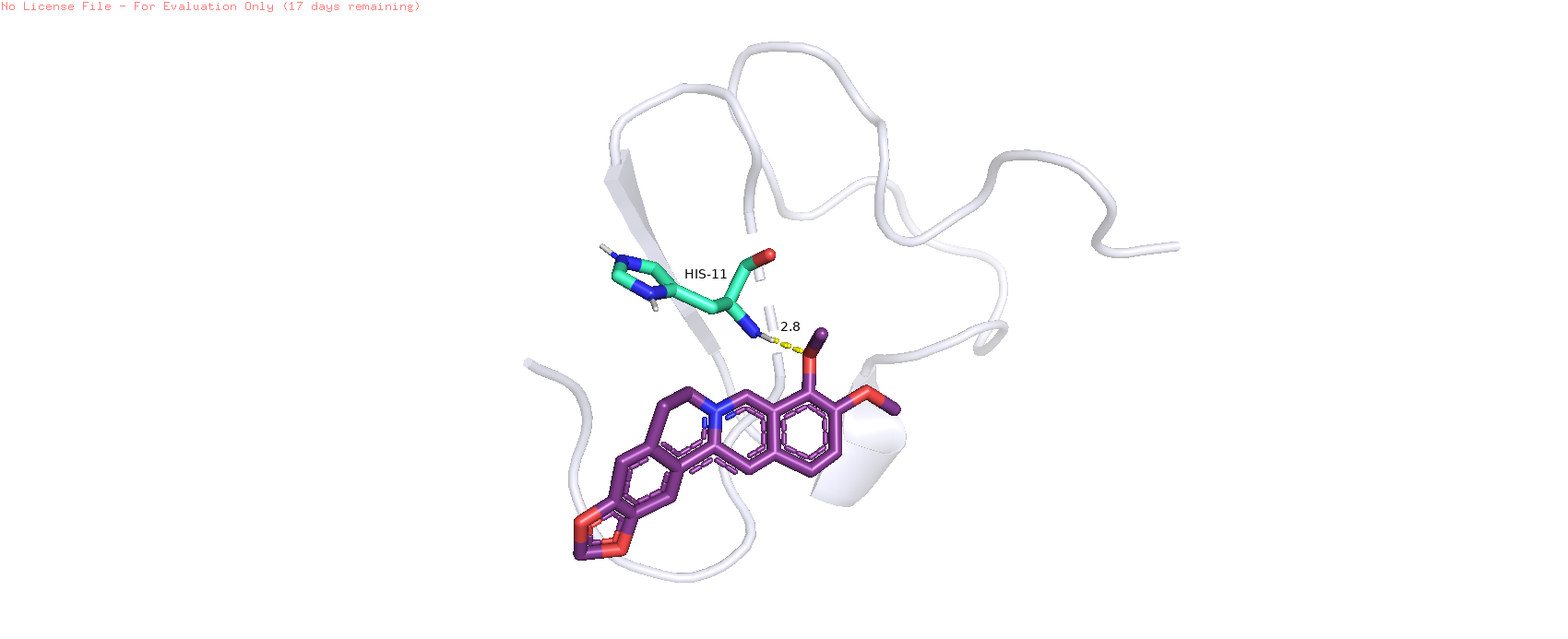

Supplement: Supplementary file 1 [file DataSheet1.ZIP › Supplementary Materials Figure S1. The binding results of BBR and metabolism-related targets/6-1AJJ-result2-ray2000.png]

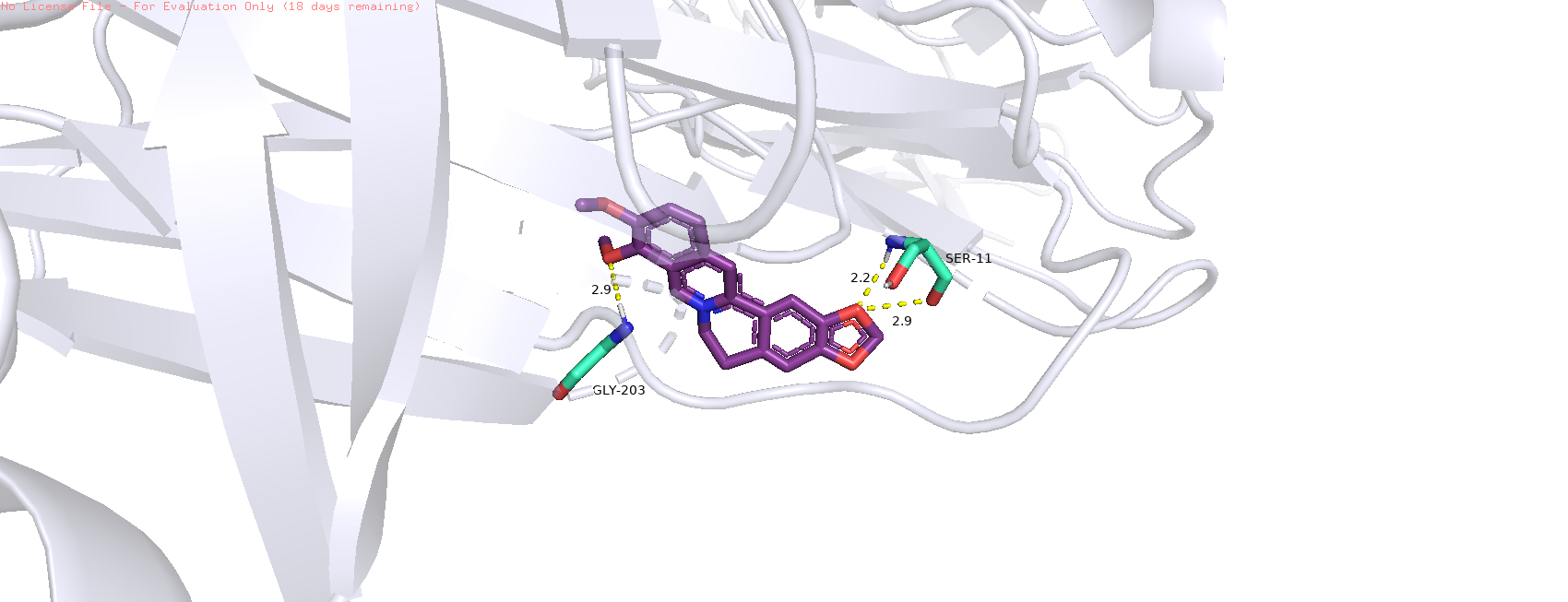

Supplement: Supplementary file 1 [file DataSheet1.ZIP › Supplementary Materials Figure S1. The binding results of BBR and metabolism-related targets/7-Fas.ray2000.png]

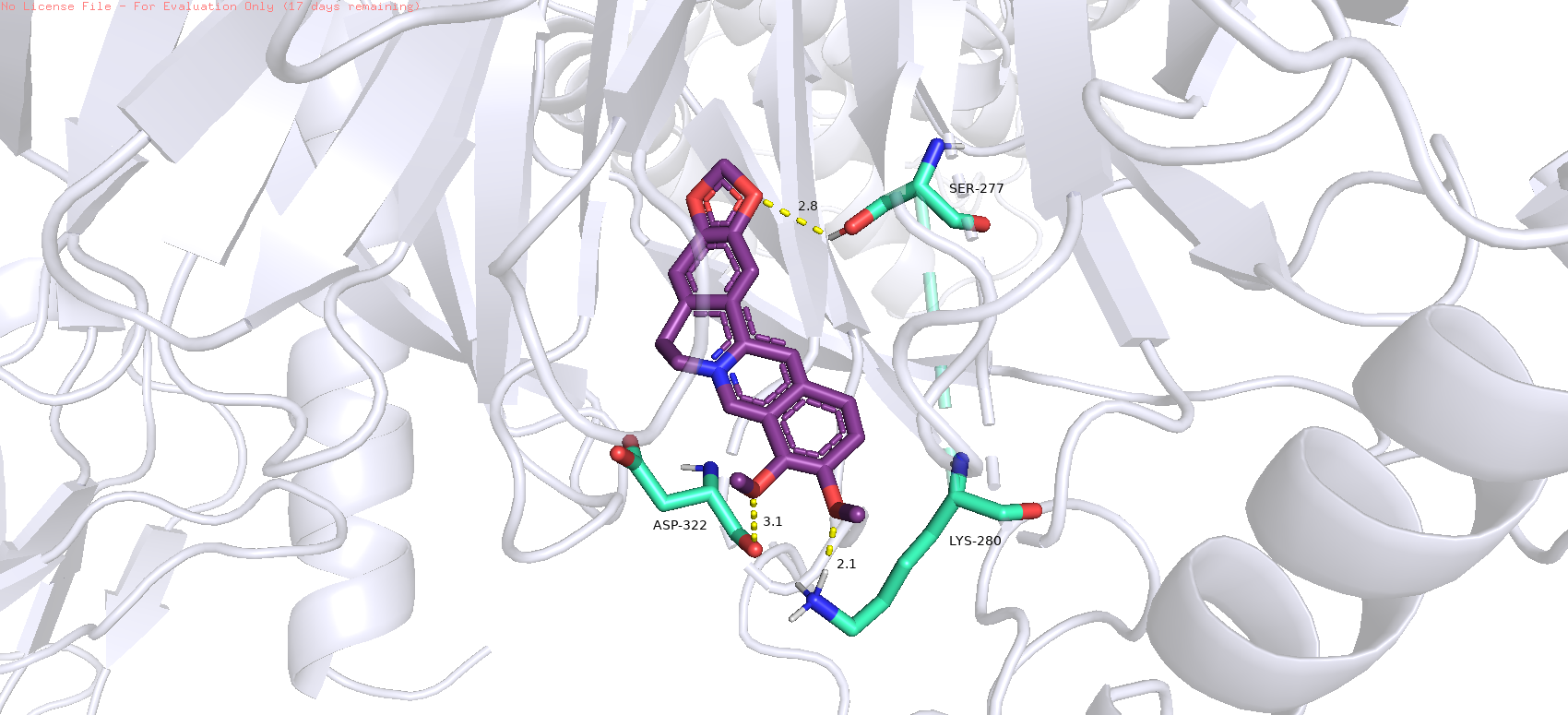

Supplement: Supplementary file 1 [file DataSheet1.ZIP › Supplementary Materials Figure S1. The binding results of BBR and metabolism-related targets/8-7XK2-ray2000.png]

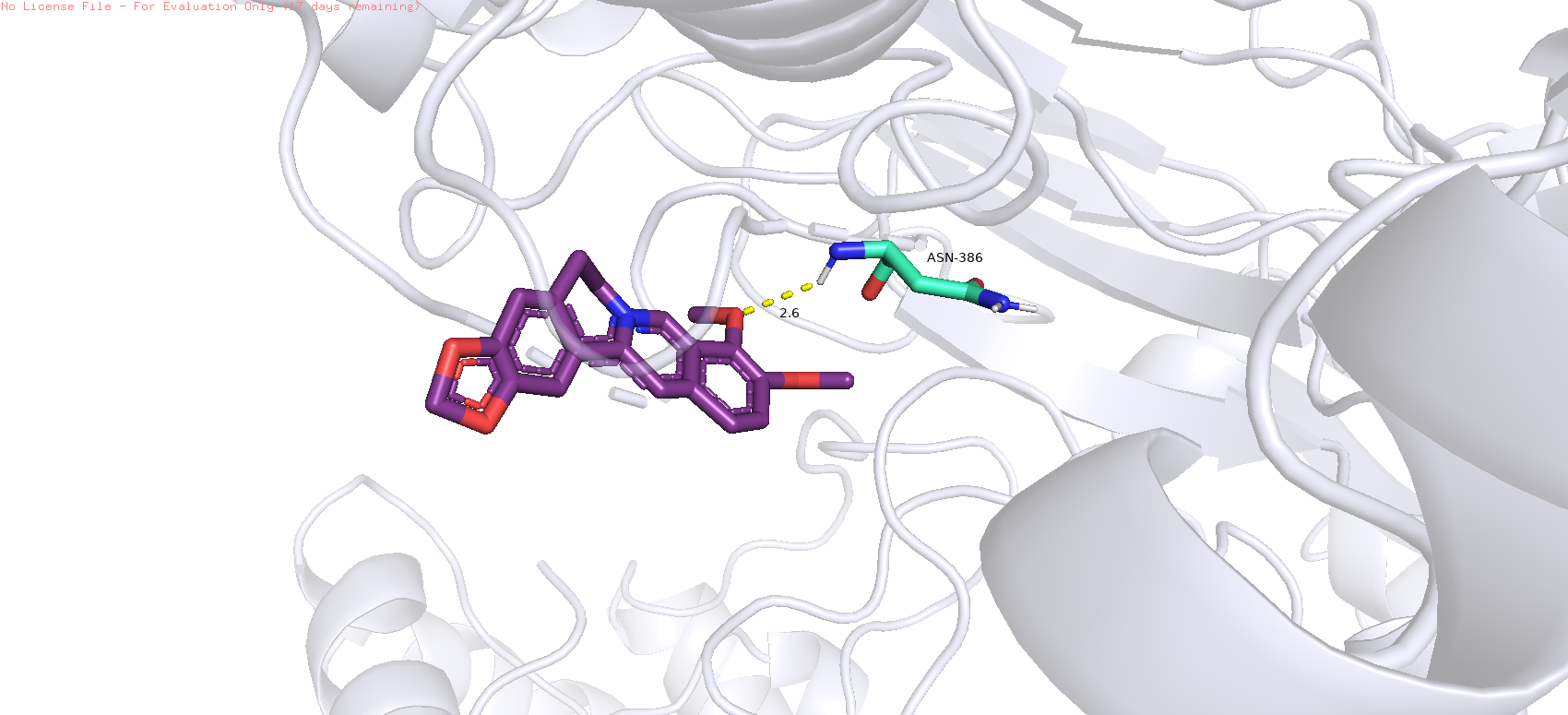

Supplement: Supplementary file 1 [file DataSheet1.ZIP › Supplementary Materials Figure S1. The binding results of BBR and metabolism-related targets/9-6FCX-ray2000.png]
